# Deciphering the epidemiological dynamics: *Toxoplasma gondii* seroprevalence in mainland China’s food animals, 2010-2023

**DOI:** 10.3389/fcimb.2024.1381537

**Published:** 2024-04-03

**Authors:** Zipeng Yang, Hao Yuan, Linchong Nie, Qingyuan Wen, Haoxin Li, Liulu Yang, Yining Song, Xun Luo, Xiu-Xiang Zhang, Zi-Guo Yuan

**Affiliations:** ^1^ Key Laboratory of Zoonosis Prevention and Control of Guangdong Province, College of Veterinary Medicine, South China Agricultural University, Guangzhou, Guangdong, China; ^2^ Kerry Rehabilitation Medicine Research Institute, Shenzhen, China; ^3^ College of Agriculture, South China Agricultural University, Guangzhou, Guangdong, China; ^4^ Key Laboratory of Zoonosis of Ministry of Agriculture and Rural Affairs, South China Agricultural University, Guangzhou, Guangdong, China

**Keywords:** *Toxoplasma gondii*, parasitology, epidemiology, public health, meta-analysis

## Abstract

**Background:**

*Toxoplasma gondii* (*T. gondii*) is a significant protozoan pathogen among food animals. Despite the threat to public health by *T. gondii* infections, there’s limited understanding of its seroprevalence and trends in food animals across mainland China. This study aimed to estimate the seroprevalence of *T. gondii* infections among swine, sheep, goats, chickens, and cattle in mainland China from 2010 to 2023.

**Methods:**

We searched cross-sectional studies published between 2010 and 2023 that reported the prevalence of *T. gondii* in food animals from databases including PubMed, Embase, Web of Science, China Biology Medicine Disc (CBM), China National Knowledge Infrastructure (CNKI), Wanfang data, and the China Science and Technology Journal Database (CQVIP). We performed subgroup analyses to explore the impact of different factors on the seroprevalence of *T. gondii*. Pooled estimates of *T. gondii* seroprevalence were calculated with a random-effects model.

**Results:**

An analysis of 184 studies involving 211985 animals revealed a *T. gondii* overall seroprevalence of 15.3% (95% CI: 13.1-17.8). Although the seroprevalence of food animals across mainland China was relatively stable from 2010 to 2023, notable variations were observed across different animal types and regions (*P* < 0.01), along with changes in geographical distribution. Sample type, detection method, animal age, and history of abortion were identified as key risk factors for *T. gondii* seroprevalence.

**Conclusion:**

The study conducted a meta-analysis on the seroprevalence of *T. gondii* in mainland China’s Food Animals from 2010 to 2023, and identified key risk factors. These findings advance our understanding of *T. gondii* infection dynamics, offering critical insights for developing control strategies and guiding public health policies.

## 1 Introduction

*Toxoplasma gondii*, a coccidian parasite, has been considered as an important zoonotic protozoan parasite in food animals, which can cause large economic losses (Dubey, 2010; Schlüter et al., 2014). It is estimated that one-third of the world population is infected with *T. gondii* (Montoya and Liesenfeld, 2004). All warm-blooded species, including people, livestock, and wildlife, serve as intermediate hosts for *T. gondii*, with cats and other felids as its only known definitive host (Dubey, 2009; Gardner et al., 2010). *T. gondii* can invade the nucleated cells of these species, where it survives and replicates (Jones and Dubey, 2012). Pregnant women and immunocompromised individuals are considered the main risk groups for *T. gondii* infection, which can cause severe health problems (Weiss and Dubey, 2009; Dubey, 2010).

Humans typically contract *T. gondii* infection by ingesting food or water contaminated with sporulated oocysts shed by the primarily infected felines or consuming undercooked or raw meat containing tissue cysts (Dubey et al., 2005). In farming environments, common food animals such as cows, sheep, and swine may contract *T. gondii* by consuming feed and water contaminated with sporulated oocysts. This infection results in the formation of cysts in their tissues, which can then be transmitted to humans if the meat is not thoroughly cooked. In addition to meat, other food items, such as milk, marine products, and vegetables can also serve as potential sources of *T. gondii* infection (Marín-García et al., 2022). Furthermore, *T. gondii* can cross the placenta, which may result in vertical transmission and congenital toxoplasmosis, especially in sheep, goats, and humans.

Over the past ten years, *T. gondii* infections in food animals have raised serious concerns among Chinese consumers (Wang et al., 2013). After the acute *T. gondii* infection, these animals typically progress to a chronic phase and carrying tissue cysts that can be transmitted to humans. Cultural factors significantly impact the differences in the prevalence of *T. gondii* between different countries, especially regarding dietary habits (Tenter et al., 2000; Robert-Gangneux and Dardé, 2012). A significant association exists between human *T. gondii* infections and consuming raw or undercooked meat (Kapperud et al., 1996; Belluco et al., 2018). In traditional Chinese cuisine, the staple foods typically consist of slowly simmered meats, rice, noodles, and various cooked vegetables, with meats having become the major and favored food in the past decades. Although most Chinese people don’t often eat undercooked meats or animal-by products, regional foods like hotpot, BBQ, and raw milk products are popular, especially with raised living standards (Li et al., 2009; Gao and Tang, 2018).

According to the National Bureau of Statistics of China (2023) (Stat. Com. of China, 2023), mainland China’s total pork, beef, mutton, and poultry output increased by 3.8% compared to the previous year, reaching 92.27 million tons in 2022, indicating an expanding preference for various animal-based foods. The consumption of different food animals is especially indicative of this rising need; chickens and ducks are the most common species among poultry, while the most commonly consumed mammals are cattle, goats, sheep, and swine. Unfortunately, conventional meat inspection methods cannot detect *T. gondii* infections, and little attention is paid to implement preventative measures within the food supply chain (Dorny et al., 2009). The absence of specific inspection strategies and standards for handling infected meat poses a potential public health concern.

There is a substantial study gap in studies on the prevalence and dynamics of *T. gondii* in food animals, especially in the last ten years, even though there has been a great deal of epidemiological research on the *T. gondii* in humans, livestock, and pets in mainland China. Significantly, existing studies primarily concentrated on evaluating the prevalence of animal *T. gondii* in a broad sense, without specifically focusing on its prevalence in food animals raised for human consumption. These studies might include diseased or already succumbed animals in their analyses, raising concern since the findings may not accurately represent the true prevalence of *T. gondii* in meat and meat products intended for human consumption.

Our research carefully excluded studies that examined sick or dead animals and instead concentrated on *T. gondii* infections in food animals produced for human consumption in mainland China between 2010 and 2023. This period has not only witnessed a substantial increase in the consumption of animal products but also brought to light the imperative need for a more profound understanding of food safety and zoonotic diseases, especially in the context of global and localized disease outbreaks. Additionally, further influenced by climate change and resultant extreme weather events in China (Li and Gu, 2020; Zhu and Fan, 2021; Cai et al., 2022; Zheng et al., 2023), these factors collectively signal the potential shifts in infectious disease dynamics. In order to address the changing health concerns in this important field, our research used meta-analysis to determine the effects of these environmental and public health changes on *T. gondii* seroprevalence.

## 2 Materials and methods

This study followed PRISMA guidelines, and the registration application for PROSPERO has been approved (ID: CRD42023437036).

### 2.1 Search strategy

To capture the majority of relevant articles, we systematically searched databases including PubMed, Embase, Web of Science, China Biology Medicine Disc (CBM), China National Knowledge Infrastructure (CNKI), Wanfang data, and the China Science and Technology journal database (CQVIP) for studies published from January 1, 2010 to October 13, 2023, with no language restrictions. The specific search steps for all databases are detailed in **Supplementary Table S1**-**4**. Additionally, we reviewed the reference lists of the analyzed studies and recent reviews.

### 2.2 Study selection and data extraction

In this study, the following inclusion criteria were considered: (1) The research subjects were common edible animals in mainland China (swine, cattle, goats, sheep, chickens). (2) Studies detected the prevalence of *Toxoplasma gondii* with the sample size and the number of positive cases. (3) Research published between 2010-2023 with a sample size of 50 or more. (4) Only serology detection methods, such as IHA, MAT, and ELISA, are acceptable. (5) Only cross-sectional study types are included.

The standard exclusion criteria included (1) Articles with reviews, abstracts, conference abstracts, and studies without raw data. (2) Studies that test multiple or mixed samples on a single animal individual. (3) Studies involving wildlife, sick animals, or dead animals. (4) Only the most comprehensive one will be included in duplicate publications or multiple articles using the same data.

Two investigators (ZP Yang and Hao Yuan) carried out the search strategy and assessed all article titles and abstracts by the eligibility standards. The information to be extracted included first author, year of publication, region, animal species, sample type, detection method, sample size, number of positive cases, sample selection, animal age, season, and location of sampling. From a food safety perspective, yaks, cattle, and dairy cows were grouped as ‘cattle’ to simplify raw data processing and enable easier comparisons among food animal species.

After selecting full articles, data was extracted individually by the two investigators using a standard form. The third investigator (ZG Yuan) resolved any assessment discrepancies with an agreement.

### 2.3 Quality assessment

The quality assessment follows the quality evaluation criteria from a similar study (Wang et al., 2021), which was based on the GRADE criteria (Guyatt et al., 2008). Four categories are scored: detection method, sample size, sample collection method, and the presence of four or more risk factors. Each category can score up to 1 point, totaling 4 points. A score of 0-1 indicates low quality, 2 is moderate quality, and 3-4 indicates high quality.

### 2.4 Statistical analysis

Pooled seroprevalence of *T. gondii* and its 95% confidence intervals (CI) were calculated by a “metaprop” command provided in Stata software (version 14.0). We utilized Cochran’s Q test and I² test for heterogeneity assessment (Higgins et al., 2003). When the results indicated no significant heterogeneity among studies (*P* ≥ 0.05, I² ≤ 50%), we employed the fixed effects model. Otherwise, we used the random effects model (*P* < 0.05, I² > 50%). Subgroup analyses were performed to explore the impact of different factors on *T. gondii* seroprevalence. The differences between the pooled seroprevalence of *T. gondii* across subgroups were analyzed using the Z-test. The funnel plots and Egger tests were used to assess publishing bias (Egger et al., 1997). The “ggplot2” R package was used to draw curves of the pooled prevalence of *T. gondii* across different years and their 95% confidence intervals. The “ComplexHeatmap” R package was used to visualize the pooled prevalence of *T. gondii* in food animals. From a food safety perspective, we include ‘cattle’ yaks, cattle, and dairy cows, facilitating consolidated comparisons among food animal species.

## 3 Results

### 3.1 Search results and eligible studies

**Figure 1:**
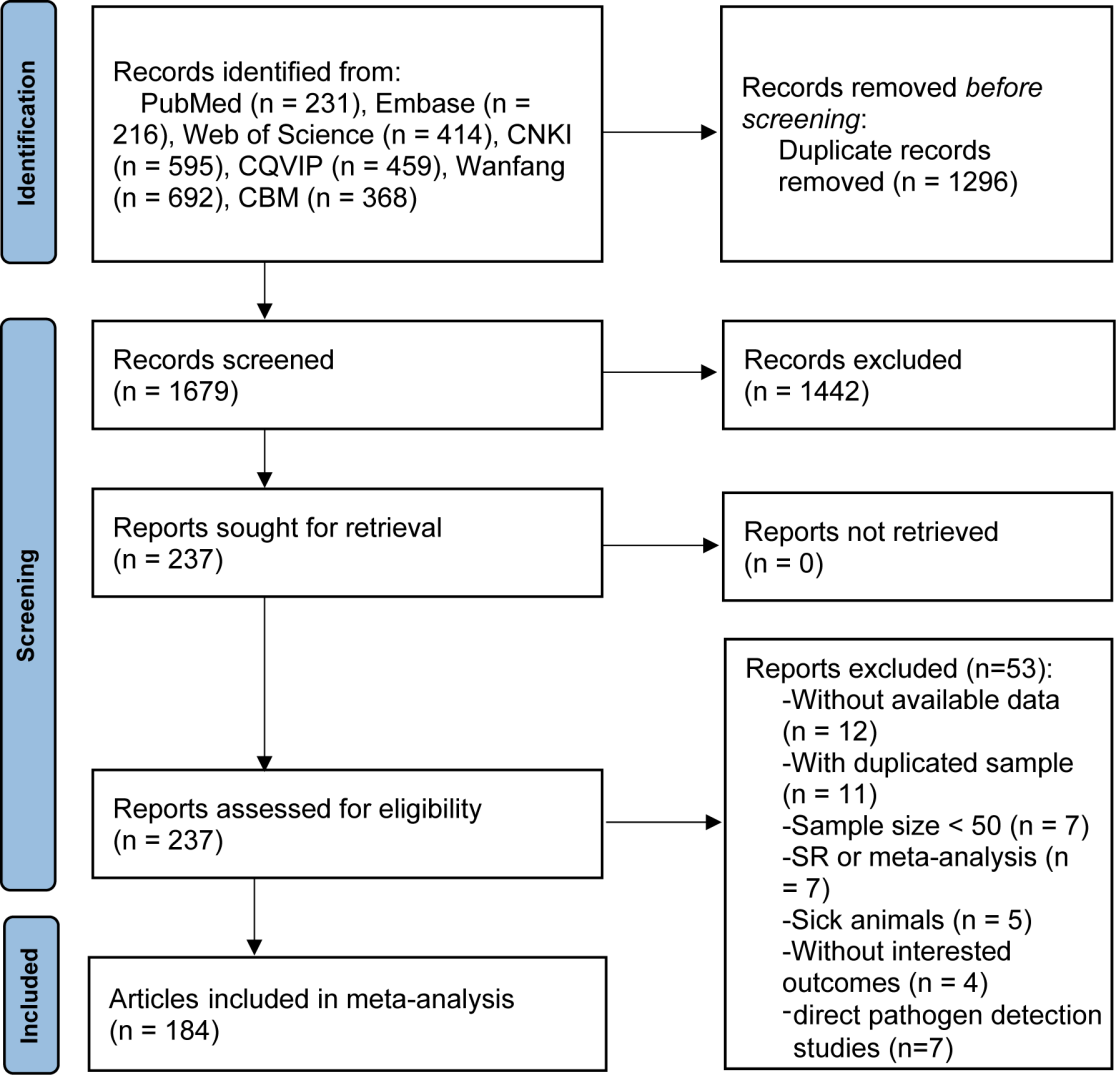
Flow-process diagram of the studies selection of meta-analysis.

**Figure 1** presents a flow-process diagram outlining the selection of relevant studies. Initially, we retrieved 2975 articles from electronic databases according to the literature search strategies. After eliminating 1296 duplicate articles, 1679 articles remained. After reviewing titles and abstracts, 1442 articles were excluded for failing to meet the inclusion criteria. Consequently, 184 articles published from 2010 to 2023, comprising 67 in English and 117 in Chinese, were deemed qualified and included in the meta-analysis. The basic characteristics of the included studies are summarized in **Supplementary Table S5**.

### 3.2 Research characteristics and quality assessment

The meta-analysis included 184 articles, which encompassed 221 studies, with total number of positive animals ranging from 50 to 30024 cases and 211,985 total tested animals. The quality assessment result of the included studies (**Supplementary Tables S5**, **S6**) revealed 154 high-quality reports, 25 medium-quality reports, and 5 low-quality reports.

### 3.3 Seroprevalence of *Toxoplasma gondii* in food animals in mainland China

**Figure 2:**
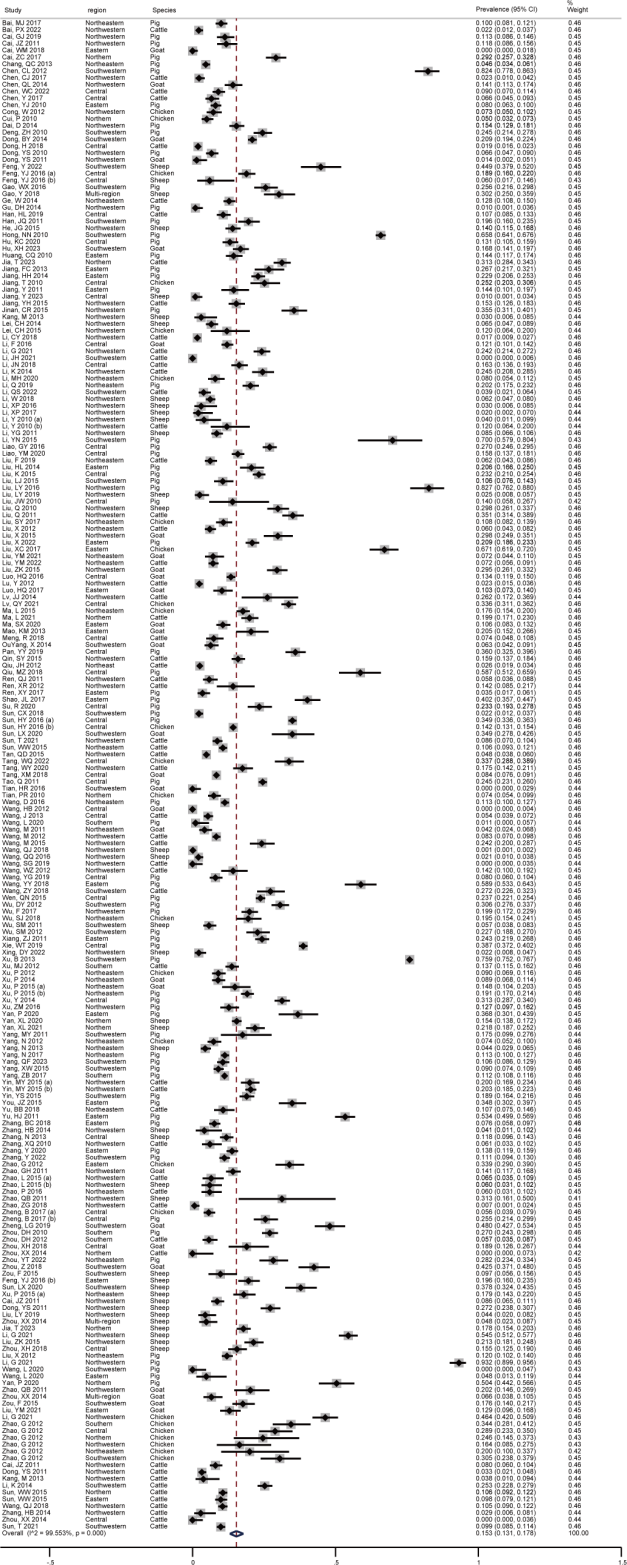
Forest plot of *T. gondii* seroprevalence of food animals in mainland China between 2010-2023.

As shown in **Figure 2**, the pooled seroprevalence of *T. gondii* infection among the swine, sheep, goats, chickens, and cattle in mainland China between 2010-2023 was 15.3% (95% CI: 13.1-17.8; 40810/211985). Subsequently, a random-effects model was used in this study (I^2^ = 99.553%, *P* < 0.001).

### 3.4 Subgroup analysis

#### 3.4.1 Seroprevalence of *Toxoplasma gondii* in different species of food animals

**Figure 3:**
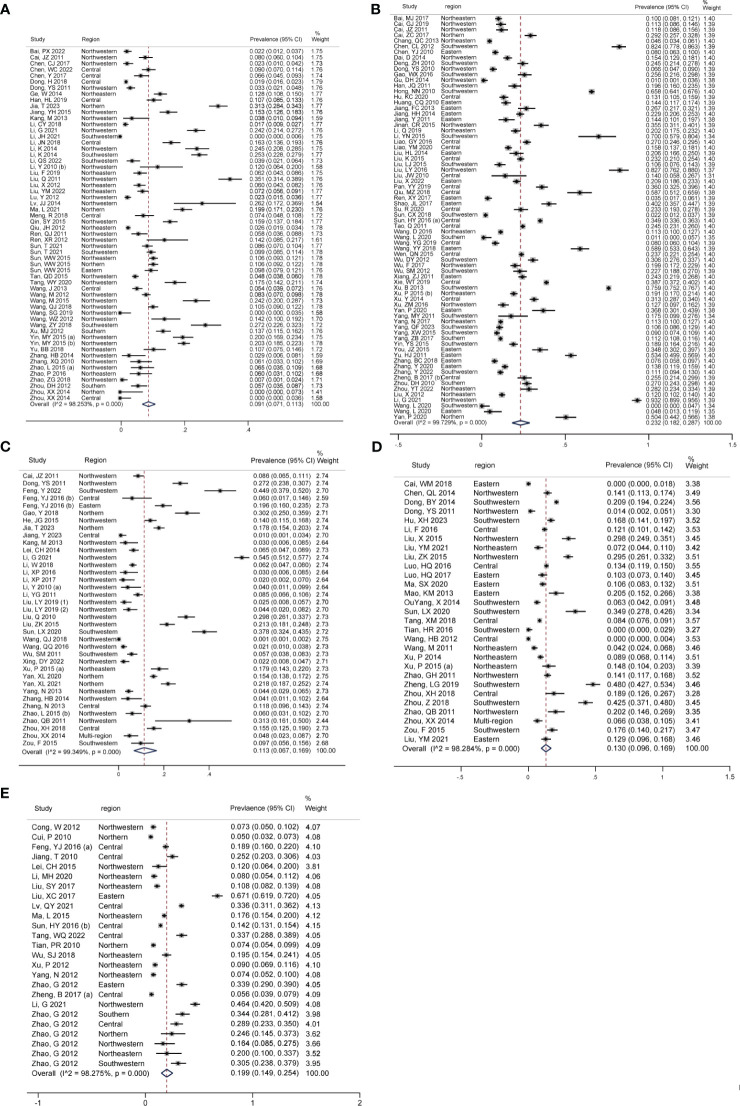
Forest plot of *T. gondii* seroprevalence in different food animals. The overall *T. gondii* seroprevalence in food animals, including **(A)** cattle, **(B)** swine, **(C)** sheep, **(D)** goats, and **(E)** chickens.

**Figures 3A–E** showed the pooled seroprevalence of *T. gondii* for swine, cattle, sheep, goats, and chickens, respectively. Specifically, there were 72 studies for swine, 58 for cattle, 37 for sheep, 29 for goats, and 25 for chickens. Among the five food animals in mainland China from 2010 to 2023, swine exhibited the highest seroprevalence of *T. gondii* at 23.2% (95% CI: 18.2, 28.7). Chickens followed this with a seroprevalence of 19.9% (95% CI: 14.9, 25.4), goats at 13.0% (95% CI: 9.6, 0.16.9), and sheep at 11.3% (95% CI: 6.7, 16.9). The lowest seroprevalence was observed in cattle, at 9.1% (95% CI: 7.1, 11.3).

Significant statistical heterogeneity was observed in the studies included for each animal: I^2^ > 50%, *P* < 0.001. Among all the food animals, there was a significant difference in *T. gondii* seroprevalence (Z = 37.505, *P* < 0.001).

#### 3.4.2 The impact of geographic factors on *Toxoplasma gondii* seroprevalence

**Figure 4:**
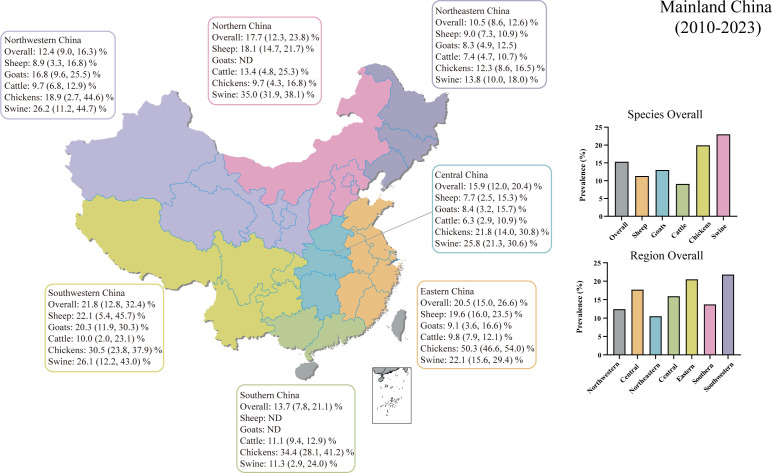
Geographical distribution map of *T. gondii* seroprevalence in sheep, goats, swine, chickens, and cattle from 2010–2023 in Mainland China. ND, not determined.

We conducted regional subgroup analyses for each animal type (**Table 1**), and the geographical distribution map of *T. gondii* seroprevalence in food animals is shown in **Figure 4**. The distribution of *T. gondii* serologic positivity rate varies across different regions in Mainland China. Notably, Southwestern China displayed the highest seroprevalence, with a pooled prevalence of 21.8% (95% CI: 12.8, 32.4). The pooled seroprevalences in other regions significantly also shape the national prevalence landscape, with Northeastern China reporting the lowest at 10.5% (95% CI: 8.6, 12.6) and the other regions showing intermediate values. Overall, there were significant differences in the *T. gondii* seroprevalence among food animals from different regions (Z = 21.131, *P* < 0.001).

**Table 1:** Subgroup analysis based on different regions.

Outcomes	No. of study	n	Positive	Prevalence (95%CI)	Heterogeneity test		Between subgroups	
					I^2^ (%)	P_H_	Z	*P* value
All								
Overall	221	211985	40810	15.3% (13.1, 17.8)	99.553	<0.001		
Region							21.131	0.004
Northeastern	29	22927	2472	10.5% (8.6, 12.6)	95.883	<0.001		
Northwestern	71	47703	5454	12.4% (9.0, 16.3)	99.272	<0.001		
Eastern	26	14229	3154	20.5% (15.0, 26.6)	98.666	<0.001		
Northern	12	8939	1633	17.7% (12.3, 23.8)	97.944	<0.001		
Southwestern	35	33991	14485	21.8% (12.8, 32.4)	99.773	<0.001		
Central	39	50809	9645	15.9% (12.0, 20.4)	99.393	<0.001		
Southern	6	32578	3854	13.7% (7.8, 21.1)	98.157	<0.001		
Multi-region	3	739	113	12.1% (1.4, 30.6)	NA	NA		
Swine								
Overall	72	103122	27873	23.2% (18.2, 28.7)	99.729	<0.001		
Region							66.241	<0.001
Northeastern	8	9553	1232	13.8% (10.0, 18.0)	96.870	<0.001		
Northwestern	10	4522	1082	26.2% (11.2, 44.7)	99.429	<0.001		
Eastern	17	10565	2435	22.1% (15.6, 29.4)	98.644	<0.001		
Northern	2	917	323	35.0% (31.9, 38.1)	NA	NA		
Southwestern	17	23197	12438	26.1% (12.2, 43.0)	99.828	<0.001		
Central	15	23227	6722	25.8% (21.3, 30.6)	98.379	<0.001		
Southern	3	31141	3641	11.3% (2.9, 24.0)	NA	NA		
Cattle								
Overall	58	44840	4633	9.1% (7.1, 11.3)	98.253	<0.001		
Region							7.225	0.301
Northeastern	8	7442	574	7.4% (4.7, 10.7)	95.681	<0.001		
Northwestern	30	18802	2209	9.7% (6.8, 12.9)	97.950	<0.001		
Eastern	1	813	80	9.8% (7.9, 12.1)	NA	NA		
Northern	4	3419	627	13.4% (4.8, 25.3)	98.483	<0.001		
Southwestern	5	4115	559	10.0% (2.0, 23.1)	99.189	<0.001		
Central	8	9024	444	6.3% (2.9, 10.9)	97.737	<0.001		
Southern	2	1225	140	11.1% (9.4, 12.9)	NA	NA		
Sheep								
Overall	37	28805	2680	11.3% (6.7, 16.9)	99.349	<0.001		
Region							48.923	<0.001
Northeastern	2	968	97	9.0% (7.3, 10.9)	NA	NA		
Northwestern	21	20724	1369	8.9% (3.3, 16.8)	99.477	<0.001		
Eastern	1	455	89	19.6% (16.0, 23.5)	NA	NA		
Northern	3	3478	601	18.1% (14.7, 21.7)	NA	NA		
Southwestern	4	1126	251	22.1% (5.4, 45.7)	98.575	<0.001		
Central	4	1558	176	7.7% (2.5, 15.3)	94.614	<0.001		
Multi-region	2	496	97	17.5% (14.2, 21.0)	NA	NA		
Goat								
Overall	29	21174	2911	13.0% (9.6, 16.9)	98.284	<0.001		
Region							14.068	0.015
Northeastern	4	1491	124	8.3% (4.9, 12.5)	85.238	<0.001		
Northwestern	6	2575	509	16.8% (9.6, 25.5)	96.433	<0.001		
Eastern	5	1756	193	9.1% (3.6, 16.6)	95.468	<0.001		
Southwestern	8	5376	1183	20.3% (11.9, 30.3)	98.238	<0.001		
Central	5	9733	886	8.4% (3.2, 15.7)	98.947	<0.001		
Multi-region	1	243	16	6.6% (3.8, 10.5)	NA	NA		
Chickens								
Overall	25	14044	2713	19.9% (14.9, 25.4)	98.275	<0.001		
Region							192.971	<0.001
Northeastern	7	3473	445	12.3% (8.6, 16.5)	91.346	<0.001		
Northwestern	4	1080	285	18.9% (2.7, 44.6)	98.62	<0.001		
Eastern	2	710	357	50.3% (46.6, 54.0)	NA	NA		
Northern	3	1125	82	9.7% (4.3, 16.8)	NA	NA		
Southwestern	1	177	54	30.5% (23.8, 37.9)	NA	NA		
Central	7	7267	1417	21.8% (14.0, 30.8)	98.388	<0.001		
Southern	1	212	73	34.4% (28.1, 41.2)	NA	NA		

^#^NA, not available; P value, difference between the subgroups.

Cattle, Dairy cows, and Yaks are collectively classified as ‘cattle’.

#### 3.4.3 Trends in the seroprevalence of *Toxoplasma gondii* in food animals in mainland China from 2010 to 2023

**Figure 5:**
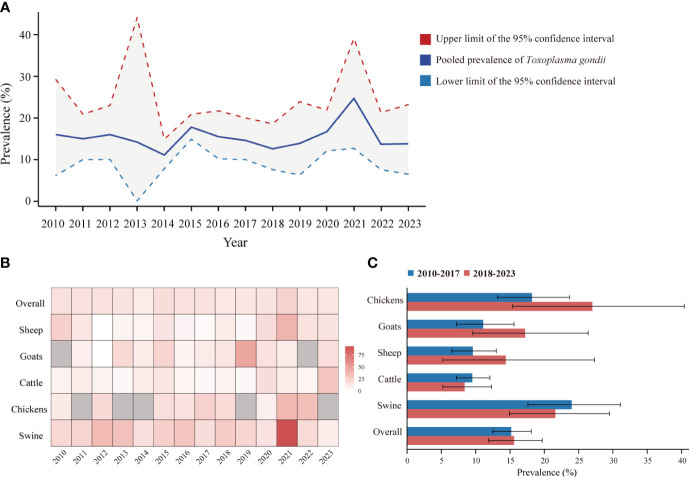
Dynamic Changes of *T. gondii* seroprevalence in sheep, goats, swine, chickens, and cattle from 2010 to 2023 in Mainland China. **(A)** Pooled seroprevalence of *T. gondii* from 2010 to 2023. **(B)** Heatmap of *T. gondii* seroprevalence changes in sheep, goats, swine, chickens, and cattle from 2010 to 2023. **(C)** *T. gondii* seroprevalence among different species of food animals between 2010 to 2017 and 2018 to 2023.

To reflect the seroprevalence trends of *T. gondii* in food animals in mainland China, as reported in studies from 2010 to 2023, we compiled and visualized the serologic positivity rates of *T. gondii* infection (**Figures 5A, B**). Overall, between 2010 and 2023, the seroprevalence of *T. gondii* remained relatively stable, with no statistically significant differences in the pooled prevalence across the years (*P* > 0.05).

The overall *T. gondii* seroprevalence for food animals was 15.2% (95% CI: 12.5, 18.1) for 2010-2017 and 15.6% (95% CI: 11.9, 19.7) for 2018-2023, with no significant difference (*P* > 0.05). Despite we observed fluctuation in *T. gondii* seroprevalence within each animal species between the periods 2010-2017 and 2018-2023, pairwise comparisons were systematically conducted between all possible subgroup pairs, revealing no significant differences (**Figure 5C**) (*P* > 0.05).

**Figure 6:**
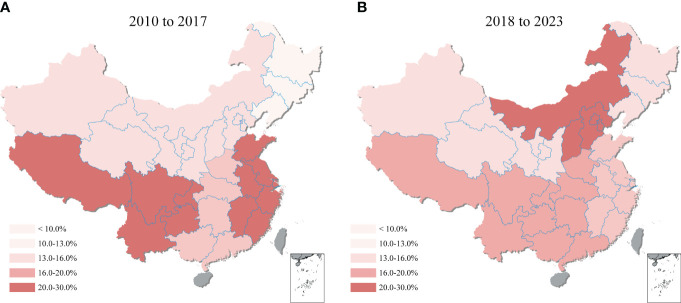
Geographical heatmap of *T. gondii* seroprevalence. The heatmap showed the seroprevalence of sheep, goats, swine, chickens, and cattle in mainland China from **(A)** 2010 to 2017 and **(B)** 2018 to 2023.

Comparing the 2010-2017 and 2018-2023 periods, shifting was observed in the regional distribution of *T. gondii* seroprevalence across mainland China (**Figure 6**). During 2010-2017, the highest seroprevalence was recorded in Southwestern China at 25.5% (95% CI: 13.8, 39.3), with Eastern China closely following at 23.4% (95% CI: 16.3, 31.3). However, the period of 2018-2023 showed Northern China rise to the highest seroprevalence at 25.2% (95% CI: 18.0, 33.1), while Southwestern China’s rate declined to the second highest at 16.7% (95% CI: 9.3, 25.6). Additionally, the region with the lowest seroprevalence changed from the Northeastern (at 9.8%, 95% CI: 7.7, 12.1) in 2010-2017 to the Northwestern (at 12.1%, 95% CI: 4.1, 23.4) in 2018-2023, highlighting the dynamic nature of *T. gondii* seroprevalence distribution over time. Details on the pooled seroprevalence across different regions for periods 2010-2017 and 2018-2023 are available in **Supplementary Table S7**.

We have completed subgroup analyses for the following factors: sample type, detection method, sample selection, quality, season, location of sampling, gender, feeding model, age, cat ownership, and history of abortion (**Table 2**). Sample type, detection method, age groups, and history of abortion were significantly associated with the seroprevalence of *T. gondii* (*P* < 0.01).

**Table 2:** Subgroup analysis based on risk factors for *Toxoplasma gondii* Infection.

Outcomes	No. of study	n	Positive	Prevalence (95%CI)	Heterogeneity test		Between subgroups	
					I^2^ (%)	P_H_	Z	*P* value
All								
Overall	221	2119856	40810	15.3% (13.1, 17.8)	99.553	<0.001		
Detection method							44.471	<0.001
MAT	23	21676	2402	13.7% (10.0, 17.9)	98.541	<0.001		
IHA	117	116198	13513	11.1% (9.4, 12.9)	98.814	<0.001		
ELISA	80	73825	24823	22.9% (17.8, 28.5)	99.663	<0.001		
LAT	1	286	72	25.2% (20.3, 30.6)	NA	NA		
Sample selection							0.964	0.326
Randomly	74	96717	13665	16.9% (13.4, 20.7)	99.515	<0.001		
Non-random	147	115268	27145	14.6% (11.7, 17.7)	99.527	<0.001		
Quality							1.457	0.483
High	188	199671	38733	15.7% (13.2, 18.4)	99.605	<0.001		
Moderate	27	11675	1899	12.6% (8.3, 17.7)	98.227	<0.001		
Low	6	639	178	19.2% (1.5, 48.9)	98.478	<0.001		
Season							1.351	0.717
Spring	36	12534	2233	14.0% (10.4, 18.0)	97.272	<0.001		
Summer	34	13652	2415	17.1% (13.0, 21.7)	97.758	<0.001		
Autumn	43	25424	3583	15.2% (11.6, 19.1)	98.571	<0.001		
Winter	27	9188	1452	14.2% (10.1,18.8)	97.212	<0.001		
Location of sampling							1.429	0.839
Farms	97	91044	15121	16.7% (13.8, 19.7)	99.273	<0.001		
Households	28	13807	3303	16.6% (9.8, 24.6)	99.271	<0.001		
Farming cooperatives	3	500	135	20.9% (1.6, 53.0)	NA	NA		
Markets	3	2059	559	13.3% (6.4, 22.2)	98.758	<0.001		
Slaughterhouses	27	27689	4691	15.6% (11.4, 20.2)	98.758	<0.001		
Gender							0.925	0.336
Female	90	63441	8520	17.5% (14.0, 21.3)	99.283	<0.001		
Male	56	12649	1934	15.0% (12.2, 18.0)	94.713	<0.001		
Feeding model							3.140	0.208
Free-range	51	22134	3601	14.4% (11.1, 18.1)	98.141	<0.001		
Semi-intensive	3	1696	150	10.5% (2.6, 22.7)	NA	NA		
Intensive	32	18966	2394	10.3% (7.4, 13.7)	97.972	<0.001		
Swine							69.681	<0.001
Piglet	22	4352	508	10.9% (7.7, 14.4)	90.941	<0.001		
Weaning pig	10	2909	311	08.1% (5.6, 11.1)	80.986	<0.001		
Growing pig	5	520	94	17.3% (10.8, 24.9)	75.848	<0.001		
Fattening pig	26	9671	1863	17.3% (13.4, 21.5)	96.288	<0.001		
Slaughter pig	4	1304	297	22.1% (17.5, 27.1)	74.338	<0.001		
Gestating sow	21	6995	2422	29.0% (21.7, 36.8)	97.73	<0.001		
Lactating sow	5	1107	362	35.5% (18.4, 54.7)	96.321	<0.001		
Replacement gilt	9	1605	561	31.8% (20.8, 43.9)	95.704	<0.001		
Breeding boar	13	1172	341	24.4% (15.6, 34.3)	90.984	<0.001		
Chicken, age (years)							42.862	<0.001
<1	4	2248	603	24.6% (19.5, 30.2)	84.762	<0.001		
1-2	2	717	341	47.6% (43.9, 51.2)	NA	NA		
Cattle, Sheep, or Goat							2.532	0.112
<=1 years	40	12494	1153	9.0% (6.6, 11.7)	94.841	<0.001		
>1 year	47	35486	3919	12.3% (9.9, 15.0)	98.047	<0.001		
Keep Cats							1.638	0.201
Yes	3	833	112	12.3% (6.6, 19.4)	NA	NA		
No	3	1469	117	8.1% (5.6, 11.0)	NA	NA		
Abortion							21.98	<0.001
Yes	4	1047	207	19.0% (14.2, 24.4)	73.059	0.011		
No	4	5487	497	7.3% (5.5, 9.4)	77.777	0.004		

^#^NA, not available; P value, difference between the subgroups.

Cattle, Dairy cows, and Yaks are collectively classified as ‘cattle’.

#### 3.4.4 Risk of bias assessment

**Figure 7:**
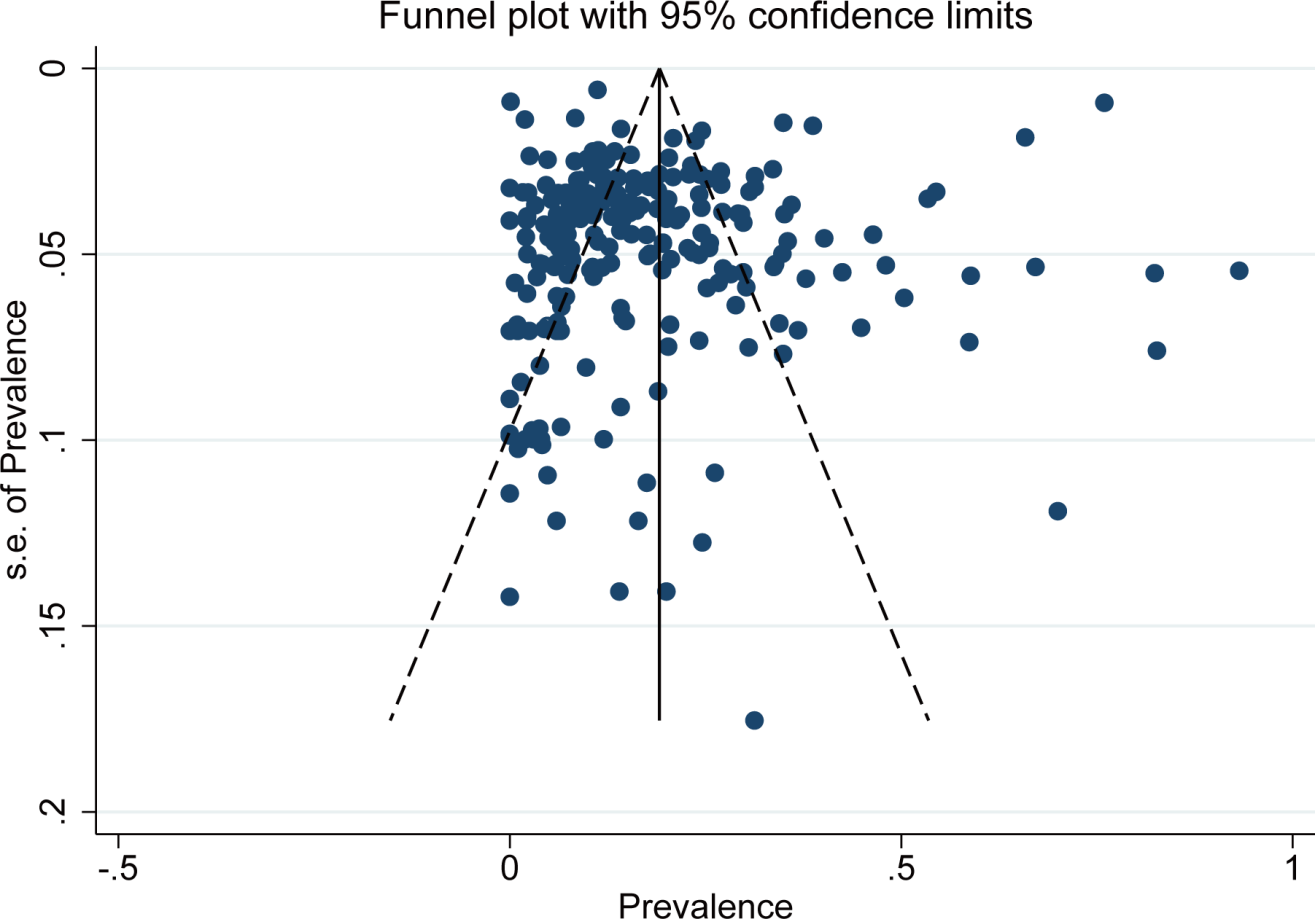
The funnel plot of *T. gondii* seroprevalence for the included studies.

The bias funnel diagram is shown in **Figure 7**. The Egger test result indicates no significant publication bias in the included studies (*P =* 0.202).

## 4 Discussion

The zoonotic illness toxoplasmosis, which affects both humans and animals, is especially dangerous for animal husbandry and public health (Montoya and Liesenfeld, 2004). The lifecycle of *T. gondii* emphasizes the role of food animals in human transmission, directly linking to the critical risk posed by consuming undercooked meat harboring *T. gondii* cysts (Dubey, 2010). Strong food safety regulations and all-encompassing approaches, including enhanced monitoring, public awareness campaigns, and more stringent food processing guidelines, are required to combat the threat and reduce the risk of *T. gondii* infection.

From 2010 to 2023, mainland China faced significant public health challenges, including COVID-19, African swine fever, and avian influenza. These challenges impacted human and animal behavior patterns (Barrett et al., 2015; Watts et al., 2017; Jalloh et al., 2020) and influenced livestock practices (Taylor et al., 2020; Brown et al., 2021; Liu et al., 2021; Petrovan et al., 2021), thereby highlighting the importance of researching *T. gondii* dynamics and understanding zoonotic diseases more broadly (Stoffel et al., 2020). Against this backdrop, gaining a deeper understanding of the epidemiology of *T. gondii* is imperative, especially in a country like China with a vast and diverse food-animal sector.

To our knowledge, this is the first meta-analysis to evaluate the seroprevalence of *T. gondii* in China’s most common food animals (cattle, sheep, goats, swine, and chickens) from 2010 to 2023. The study’s findings offer valuable insights into *T. gondii* seroprevalence, crucial for public health experts and policymakers for designing and implementing effective control strategies, particularly in food safety and animal disease management. Our research lays the groundwork for further exploration into *T. gondii*’s transmission mechanisms and preventive measures. Additionally, it may serve as a reference point for other researchers delving deeper into this field, enhancing the understanding of the global epidemiology of toxoplasmosis.

The seroprevalence of *T. gondii* among food animals in mainland China from 2010 to 2023 was 15.3%, which is lower than the reported rates for worldwide livestock and poultry between 2000-2019 (28.3%) (Hajimohammadi et al., 2022), and the seroprevalence observed in China’s food animals between 2000-2017 (23.7%) (Dong et al., 2018), and China’s pigs from 1990-2017 (24%) (Foroutan et al., 2019). The difference may be attributed to the differences in the number of studies included, methodological differences in studies, sample selection biases, and notably, the influence of evolving environmental and societal factors over time. Despite the absence of specific food safety measures targeting *T. gondii* in China, it’s crucial to consider that environmental and societal factors influence *T. gondii* transmission by altering critical conditions for its survival, such as humidity and temperature (Robert-Gangneux and Dardé, 2012). Additionally, a series of epidemic control measures may weaken the transmission of *T. gondii*. For instance, wildlife protection measures reduce contact between natural hosts of *T. gondii* and livestock, while closed management and all-in-all-out policies could decrease the spread of pathogens within livestock populations.

To better understand the changes in *T. gondii* seroprevalence, we divided our data into two periods: 2010 to 2017 and 2018 to 2023. Although the seroprevalence remained relatively stable between 2010-2023, significant regional differences in distribution between different periods were observed. The China Meteorological Administration’s 2023 Blue Book (Press, 2023) reveals considerable climate changes across various regions of China since 2010, including an increase in average annual precipitation and a continuous rise in temperatures from 2015 to 2022, ranking among the hottest eight years since 1961. Additionally, it highlights the rise in extreme weather events, which has raised the climate risk index considerably. The regional variations in these environmental factors may have influenced the distribution of *T. gondii*. Since 2018, compared to the previous period, outbreaks of COVID-19 and African Swine Fever have profoundly affected public health security in mainland China, likely leading to substantial changes in animal management and transportation strategies, such as enhanced wildlife protection measures, stricter animal transport restrictions, and quarantine measures. In addition, the impact of the disease outbreak has shifted consumer preferences towards consuming locally or regionally-produced meat. These comprehensive factors could influence the *T. gondii* seroprevalence and its distribution in food animals across specific regions.

Although the seroprevalence of *T. gondii* within each species of food animals shows no statistically significant differences across the two time periods, the observed fluctuations should not be overlooked. These variations, while not currently significant, have the potential to manifest more clearly in studies with larger sample sizes or longer durations, highlighting the necessity for ongoing surveillance. Effective and targeted prevention and control techniques for various locations and species must be implemented in future public health policies and animal disease management plans.

We conducted extensive subgroup analyses to dissect the risk factors of *T. gondii* infection. Animal species, geographical location, year-to-year variations, sample type, detection method, age groups, and history of abortion are identified as key risk factors influencing the *T. gondii* seroprevalence. China is one of the countries with the highest biodiversity in the world, boasting various landscapes and animal populations. The variability in *T. gondii* prevalence among different species can be attributed to differences in susceptibility, which is largely influenced by the host immune system and how it is modulated by parasitic factors (Innes, 1997; Mukhopadhyay et al., 2020). Among the food animal species we monitored, swine and cattle had the highest and lowest rates of *T. gondii* serological positivity, respectively, while goats and sheep had intermediate rates, also supported by Pan et al (Pan et al., 2017). Significantly, our findings reveal a rising trend of *T. gondii* prevalence in chickens, particularly in the southern regions, which marks an emerging concern when compared to past reviews (Pan et al., 2017; Wang et al., 2021). However, it should be noted that the scarcity of available data for certain species might lead to discrepancies between our results and the actual seroprevalence, particularly when examining specific regions. Accurately determining *T. gondii* prevalence for various species, especially in regions where studies are limited, necessitates increased involvement from researchers across diverse regions in future research efforts. While data from 2010 to 2023 indicate a stability of *T. gondii* seroprevalence, year-specific fluctuations in pooled prevalence highlight the need for ongoing monitoring, which is particularly evident between certain years. Additionally, the 95% confidence interval variability for the pooled prevalence, whether within a single year or across different years, underscores the influence of various factors on disease prevalence at different times and locations. This variability further supports the necessity of ongoing monitoring of these local dynamics.

We observed that swine and chickens of greater age, specifically adults, tend to have higher positivity of *T. gondii*, suggesting the possibility of widespread chronic infections due to breeding conditions and environmental factors in these species. The subgroup analyses suggest that replacement gilts, lactating sows, and gestating sows exhibit the highest prevalence of *T. gondii* among different types of pigs. Vertical transmission of *T. gondii* mainly occurs during the initial acquisition of the infection by immunologically naive pregnant individuals (Tenter et al., 2000). Considering our findings of lowest seroprevalence in piglets and weaning pigs, we infer that most gestating sows are likely infected with *T. gondii* before pregnancy, providing a certain level of protection to the piglets. To control the spread of *T. gondii*, breeding institutions should focus on closely monitoring and screening these high-risk groups and strictly manage the environment of pig farms and pens. While a similar trend is observed in chickens of different ages, research on this aspect is extremely scarce. Therefore, future research should focus more on this issue, especially on whether chickens of different ages exhibit significantly different prevalences of *T. gondii*.

*T. gondii* infection can lead to abortions or fetal developmental disorders in small ruminants and pregnant women (Dubey, 2010). Despite the subgroup analysis on the history of abortion involving only a limited number of studies, evidence indicates a strong correlation between *T. gondii* infection and the incidence of miscarriages. In China, the seroprevalence of *T. gondii* among pregnant entities is significantly higher in animals than in women, with pigs at 24% and chickens and ruminants at 20%, in contrast to 5.0% or less in pregnant women (Deng et al., 2018). Additionally, variations in *T. gondii* seroprevalence were observed based on different detection methods. Although there are recognized methods for detecting *T. gondii*, standardization of detection methods in the food industry is still needed (Koutsoumanis et al., 2018; Marín-García et al., 2022). Given the current limitations in detecting infected animals before slaughter, including the economic and practical infeasibility of employing the most sensitive methods like PCR on a large scale, it’s crucial to explore and adopt a multifaceted approach. We advocate for integrating feasible testing strategies within a broader inspection and quarantine framework, aiming to enhance food safety by mitigating risks associated with meat and related products from edible animals.

To reduce the risks posed by *T. gondii* to public health and food safety, governments and researchers need to monitor its prevalence trends and craft region-specific prevention strategies. Establishing standardized detection methods for this parasite, supported nationally, is critical for accurate toxoplasmosis diagnosis. We encourage quarantine departments to periodically employ standardized methods for monitoring *T. gondii* in food animals to track its prevalence. Furthermore, incorporating *T. gondii* detection into the pre-slaughter quarantine of livestock following the monitoring of localized outbreaks is a measure to ensure public health.

In summary, this study comprehensively analyzes *T. gondii* seroprevalence in China’s food animals based on extensive research and a substantial, high-quality dataset, ensuring reliable results. It paves the way for further investigations into *T. gondii*’s transmission and prevention, offering valuable insights for researchers and advancing global understanding of toxoplasmosis epidemiology.

However, our study has limitations. Firstly, our results demonstrate a high degree of heterogeneity, the sources we could not identify through sensitivity or subgroup analysis. Secondly, due to data limitations, our study could not account for the variability in sensitivity and specificity among detection methods used in the original research, leading to uncertainty or variance in seroprevalence rates. Such variability introduces additional uncertainty to the outcomes of this study. Therefore, future research should establish universal detection methods and standards, especially for tailored measures in different regions. Thirdly, some articles lack critical information such as age, gender, breeding methods, cat ownership, and history of abortion, which limits the ability to conduct more detailed analyses on specific subgroups. Lastly, caution is required when generalizing these findings globally since all included studies are conducted in China. This geographical limitation suggests a need for broader, international research to validate and extend our findings.

## 5 Conclusion

In mainland China, the seroprevalence of *T. gondii* in food animals was 15.3% (95% CI: 13.1-17.8) between 2010 and 2023, with observed species and regional variations. Although the overall trend of *T. gondii* seroprevalence remained stable, a notable shift was observed across different areas. Thus, it is advised to implement and uphold stringent preventative and control measures, such as increased monitoring and better food safety procedures.

## Data availability statement

The original contributions presented in the study are included in the article/**Supplementary Material**. Further inquiries can be directed to the corresponding authors.

## Author contributions

ZY: Writing – review & editing, Conceptualization, Formal analysis, Funding acquisition, Methodology, Software, Supervision, Visualization. HY: Investigation, Methodology, Writing – review & editing. LN: Formal analysis, Methodology, Writing – review & editing. QW: Writing – review & editing, Visualization. HL: Formal analysis, Methodology, Writing – review & editing. LY: Formal analysis, Methodology, Writing – review & editing. YS: Data curation, Methodology, Writing – review & editing. XL: Visualization, Writing – review & editing. XZ: Conceptualization, Supervision, Writing – original draft, Writing – review & editing. Z-GY: Conceptualization, Supervision, Writing – review & editing, Resources, Writing – original draft.
