# Supplementary material for: Deciphering the epidemiological dynamics: Toxoplasma gondii seroprevalence in mainland China’s food animals, 2010-2023
Source: Front Cell Infect Microbiol. 2024 Apr 3;14:1381537. doi: 10.3389/fcimb.2024.1381537 (PMC11021580; doi:10.3389/fcimb.2024.1381537)
Supplement: Supplementary file 1 [file Table_1.docx]

The retrieval time: October 13, 2023

**Table S1. The search steps and results on the PubMed database.**

| Search | Query | Items found |
| --- | --- | --- |
| #1 | ("toxoplasma"[MeSH Terms] OR "toxoplasma"[All Fields] OR "toxoplasmas"[All Fields] OR "toxoplasma gondii"[All Fields]) | 23182 |
| #2 | ("china"[MeSH Terms] OR "china"[All Fields] OR "china's"[All Fields] OR "people's republic of china"[All Fields] OR ("mainland"[All Fields] AND "china"[All Fields])) | 2746101 |
| #3 | "epidemiology"[MeSH Subheading] OR "epidemiology"[All Fields] OR "prevalence"[All Fields] OR "prevalence"[MeSH Terms] OR "prevalance"[All Fields] OR "prevalences"[All Fields] OR ("positive"[All Fields] OR "positively"[All Fields]) OR "infect*"[All Fields] OR ("seroepidemiologic studies"[MeSH Terms] OR "seroprevalence"[All Fields] OR "seroprevalences"[All Fields] OR "seroprevalance"[All Fields] OR "seroprevalances"[All Fields] OR "seroprevalency"[All Fields] OR "seroprevalent"[All Fields]) OR ("epidemiologies"[All Fields] OR "epidemiology"[MeSH Terms]) | 7252502 |
| #4 | ("swine"[MeSH Terms] OR "swine"[All Fields] OR "pig"[All Fields]) OR ("cattle"[MeSH Terms] OR "cattle"[All Fields] OR "cattles"[All Fields] OR "yaks"[All Fields] OR "cow"[All Fields]) OR ("goats"[MeSH Terms] OR "goats"[All Fields] OR "goat"[All Fields]) OR ("sheep"[MeSH Terms] OR "sheep"[All Fields] OR "sheeps"[All Fields] OR "domestic sheep"[All Fields]) OR ("chickens"[MeSH Terms] OR "chickens"[All Fields] OR "chicken"[All Fields]) | 1029207 |
| #5 | #1 AND #2 AND #3 AND #4 | 251 |

**Table S2. The search steps and results on the Embase database.**

| Search | Query | Items found |
| --- | --- | --- |
| #1 | ('toxoplasma'/exp OR toxoplasma OR toxoplasmas OR 'toxoplasma gondii'/exp OR 'toxoplasma gondii') | 23658 |
| #2 | ('china'/exp OR china OR 'mainland china') | 2402958 |
| #3 | ('prevalence'/exp OR prevalence OR positive OR infect* OR 'seroprevalence'/exp OR seroprevalence OR 'epidemiology'/exp OR epidemiology) | 8490036 |
| #4 | ('swine'/exp OR swine OR 'pig'/exp OR pig OR 'cattle'/exp OR cattle OR 'goat'/exp OR goat OR 'sheep'/exp OR sheep OR 'chicken'/exp OR chicken OR 'yaks'/exp OR yaks OR 'cow'/exp OR cow) | 693394 |
| #5 | #1 AND #2 AND #3 AND #4 | 216 |

**Table S3. The search steps and results on the Web of Science database.**

| Search | Query | Items found |
| --- | --- | --- |
| #1 | Toxoplasma OR Toxoplasmas OR “Toxoplasma gondii” (All Fields) | 19277 |
| #2 | China OR “People's Republic of China” OR “Mainland China” (All Fields) | 6523423 |
| #3 | prevalence OR positive OR infect* OR Seroprevalence OR Epidemiology (All Fields) | 4986864 |
| #4 | swine OR Pig OR cattle OR goat OR sheep OR chicken OR Yaks OR cow (All Fields) | 600940 |
| #5 | #1 AND #2 AND #3 AND #4 | 414 |

**Table S4. The search steps and results on four Chinese databases.**

| Database | Query | Items found |
| --- | --- | --- |
| WANFANG | 题名或关键词:(弓形虫) AND (全部:(感染) OR 全部:(阳性) ) AND (题名或关键词:(猪) OR 题名或关键词:(牛) OR 题名或关键词:(羊) OR 题名或关键词:(鸡)) AND (全部:("流行病学") OR 全部:("血清学") OR 题名或关键词:("调查") OR 全部:(率)) | 692 |
| CNKI | TI="弓形虫" AND (TKA ="感染" OR TKA = "阳性") AND (TKA =猪 OR TKA =牛 OR TKA =羊 OR TKA =鸡) AND (TKA ="流行病学" OR TKA ="血清学" OR TKA ="调查" OR TKA = 率) | 595 |
| 维普 | M="弓形虫" AND (U ="感染" OR U ="阳性") AND (U ="猪" OR U ="牛" OR U ="羊" OR U ="鸡") AND (U ="流行病学" OR U ="血清学" OR U ="调查" OR U ="率") | 459 |
| SinoMed | "弓形虫"[中文标题:智能] AND ("感染"[常用字段:智能] OR "阳性"[常用字段:智能]) AND ("猪"[常用字段:智能] OR "牛"[常用字段:智能] OR "羊"[常用字段:智能] OR "鸡"[常用字段:智能]) | 368 |

^#^The query was searched in Chinese
