# Supplementary material for: Deciphering the epidemiological dynamics: Toxoplasma gondii seroprevalence in mainland China’s food animals, 2010-2023
Source: Front Cell Infect Microbiol. 2024 Apr 3;14:1381537. doi: 10.3389/fcimb.2024.1381537 (PMC11021580; doi:10.3389/fcimb.2024.1381537)
Supplement: Supplementary file 2 [file Table_2.docx]

**Table S5. Basic characteristics of included studies.**

| ID | Study | Species | Region | Sample type | Detection method | Quality score | Quality | Total samples | Positive samples |
| --- | --- | --- | --- | --- | --- | --- | --- | --- | --- |
| 1 | Bai, MJ 2017 | Swine | Northeastern | Blood, Serum | MAT | 4 | High | 882 | 88 |
| 2 | Bai, PX 2022 | Cattle | Northwestern | Blood, Serum | IHA | 3 | High | 600 | 13 |
| 3 | Cai, GJ 2019 | Swine | Northwestern | Blood, Serum | IHA | 3 | High | 460 | 52 |
| 4 | Cai, JZ 2011 | Swine | Northwestern | Blood, Serum | IHA | 3 | High | 348 | 41 |
|  |  | Sheep |  |  |  |  |  | 629 | 54 |
|  |  | Cattle |  |  |  |  |  | 635 | 51 |
| 5 | Cai, WM 2018 | Goat | Eastern | Blood, Serum | IHA | 3 | High | 200 | 0 |
| 6 | Cai, ZC 2017 | Swine | Northern | Blood, Serum | IHA | 2 | Moderate | 655 | 191 |
| 7 | Chang, QC 2013 | Swine | Northeastern | Blood, Serum | IHA | 3 | High | 1014 | 47 |
| 8 | Chen, CL 2012 | Swine | Southwestern | Blood, Serum | ELISA | 3 | High | 329 | 271 |
| 9 | Chen, CJ 2017 | Cattle | Northwestern | Blood, Serum | IHA | 3 | High | 400 | 9 |
| 10 | Chen, QL 2014 | Goat | Northwestern | Blood, Serum | ELISA | 3 | High | 524 | 74 |
| 11 | Chen, WC 2022 | Cattle | Central | Blood, Serum | IHA | 4 | High | 710 | 64 |
| 12 | Chen, Y 2017 | Cattle | Central | Blood, Serum | IHA | 3 | High | 454 | 30 |
| 13 | Chen, YJ 2010 | Swine | Eastern | Blood, Serum | IHA | 3 | High | 882 | 71 |
| 14 | Cong, W 2012 | Chicken | Northwestern | Blood, Serum | MAT | 3 | High | 413 | 30 |
| 15 | Cui, P 2010 | Chicken | Northern | Blood, Serum | IHA | 3 | High | 484 | 24 |
| 16 | Dai, D 2014 | Swine | Northwestern | Blood, Serum | ELISA | 3 | High | 768 | 118 |
| 17 | Deng, ZH 2010 | Swine | Southwestern | Blood, Serum | IHA | 2 | Moderate | 711 | 174 |
| 18 | Dong, BY 2014 | Goat | Southwestern | Blood, Serum | IHA | 3 | High | 2840 | 593 |
| 19 | Dong, H 2018 | Cattle | Central | Blood, Serum | MAT | 3 | High | 5292 | 102 |
| 20 | Dong, YS 2010 | Swine | Northwestern | Blood, Serum | IHA | 3 | High | 557 | 37 |
| 21 | Dong, YS 2011 | Goat | Northwestern | Blood, Serum | IHA | 3 | High | 140 | 2 |
|  |  | Cattle |  |  |  |  |  | 738 | 24 |
|  |  | Sheep |  |  |  |  |  | 670 | 182 |
| 22 | Feng, Y 2022 | Sheep | Southwestern | Blood, Serum | IHA | 3 | High | 205 | 92 |
| 23 | Feng, YJ 2016 (a) | Chicken | Central | Blood, Serum | MAT | 2 | Moderate | 700 | 132 |
| 24 | Feng, YJ 2016 (b) | Sheep | Central | Blood, Serum | MAT | 3 | High | 67 | 4 |
|  |  | Sheep | Eastern |  |  |  |  | 455 | 89 |
| 25 | Gao, WX 2016 | Swine | Southwestern | Blood, Serum | ELISA | 3 | High | 454 | 116 |
| 26 | Gao, Y 2018 | Sheep | Northern | Blood, Serum | IHA | 4 | High | 288 | 87 |
| 27 | Ge, W 2014 | Cattle | Northeastern | Blood, Serum | ELISA | 3 | High | 1040 | 133 |
| 28 | Gu, DH 2014 | Swine | Northwestern | Blood, Serum | ELISA | 3 | High | 200 | 2 |
| 29 | Han, HL 2019 | Cattle | Central | Blood, Serum | IHA | 3 | High | 672 | 72 |
| 30 | Han, JQ 2011 | Swine | Southwestern | Blood, Serum | IHA | 3 | High | 450 | 88 |
| 31 | He, JG 2015 | Sheep | Northwestern | Blood, Serum | IHA | 3 | High | 707 | 99 |
| 32 | Hong, NN 2010 | Swine | Southwestern | Blood, Serum | ELISA | 3 | High | 2906 | 1913 |
| 33 | Hu, KC 2020 | Swine | Central | Blood, Serum | ELISA | 3 | High | 628 | 82 |
| 34 | Hu, XH 2023 | Goat | Southwestern | Blood, Serum | MAT | 3 | High | 734 | 123 |
| 35 | Huang, CQ 2010 | Swine | Eastern | Blood, Serum | IHA | 3 | High | 605 | 87 |
| 36 | Jia, T 2023 | Cattle | Northern | Blood, Serum | ELISA | 3 | High | 978 | 306 |
|  |  | Sheep |  |  |  |  |  | 984 | 175 |
| 37 | Jiang, FC 2013 | Swine | Eastern | Blood, Serum | ELISA | 3 | High | 300 | 80 |
| 38 | Jiang, HH 2014 | Swine | Eastern | Blood, Serum | IHA | 3 | High | 1232 | 282 |
| 39 | Jiang, T 2010 | Chicken | Central | Blood, Serum | LAT | 3 | High | 286 | 72 |
| 40 | Jiang, Y 2011 | Swine | Eastern | Blood, Serum | IHA | 2 | Moderate | 222 | 32 |
| 41 | Jiang, Y 2023 | Sheep | Central | Tissue | MAT | 2 | Moderate | 210 | 2 |
| 42 | Jiang, YH 2015 | Cattle | Northwestern | Blood, Serum | IHA | 3 | High | 660 | 101 |
| 43 | Jinan, CR 2015 | Swine | Northwestern | Blood, Serum | ELISA | 3 | High | 462 | 164 |
| 44 | Kang, M 2013 | Sheep | Northwestern | Blood, Serum | IHA | 2 | Moderate | 100 | 3 |
|  |  | Cattle |  |  |  |  |  | 106 | 4 |
| 45 | Lei, CH 2014 | Sheep | Northwestern | Blood, Serum | IHA | 3 | High | 565 | 37 |
| 46 | Lei, CH 2015 | Chicken | Northwestern | Blood, Serum | IHA | 2 | Moderate | 100 | 12 |
| 47 | Li, CY 2018 | Cattle | Northwestern | Blood, Serum | IHA | 3 | High | 900 | 15 |
| 48 | Li, F 2016 | Goat | Central | Blood, Serum | IHA | 4 | High | 1028 | 124 |
| 49 | Li, G 2021 | Cattle | Northwestern | Blood, Serum | ELISA | 3 | High | 868 | 210 |
|  |  | Swine |  |  |  |  |  | 337 | 314 |
|  |  | Chicken |  |  |  |  |  | 500 | 232 |
|  |  | Sheep |  |  |  |  |  | 907 | 494 |
| 50 | Li, JH 2021 | Cattle | Southwestern | Blood, Serum | ELISA | 2 | Moderate | 596 | 0 |
| 51 | Li, JN 2018 | Cattle | Central | Blood, Serum | IHA | 4 | High | 670 | 109 |
| 52 | Li, K 2014 | Cattle | Northwestern | Blood, Serum | IHA | 3 | High | 510 | 125 |
|  |  | Cattle | Southwestern |  |  |  |  | 1128 | 285 |
| 53 | Li, MH 2020 | Chicken | Northeastern | Blood, Serum | IHA | 4 | High | 377 | 30 |
| 54 | Li, Q 2019 | Swine | Northeastern | Blood, Serum | IHA | 4 | High | 806 | 163 |
| 55 | Li, QS 2022 | Cattle | Southwestern | Blood, Serum | ELISA | 3 | High | 363 | 14 |
| 56 | Li, W 2018 | Sheep | Northwestern | Blood, Serum | IHA | 3 | High | 890 | 55 |
| 57 | Li, XP 2016 | Sheep | Northwestern | Blood, Serum | IHA | 2 | Moderate | 100 | 3 |
| 58 | Li, XP 2017 | Sheep | Northwestern | Blood, Serum | IHA | 2 | Moderate | 100 | 2 |
| 59 | Li, Y 2010 (a) | Sheep | Northwestern | Blood, Serum | IHA | 2 | Moderate | 100 | 4 |
| 60 | Li, Y 2010 (b) | Cattle | Northwestern | Blood, Serum | IHA | 2 | Moderate | 100 | 12 |
| 61 | Li, YG 2011 | Sheep | Northwestern | Blood, Serum | IHA | 3 | High | 781 | 66 |
| 62 | Li, YN 2015 | Swine | Southwestern | Blood, Serum | ELISA | 1 | Low | 70 | 49 |
| 63 | Liao, GY 2016 | Swine | Central | Blood, Serum | ELISA | 4 | High | 1302 | 351 |
| 64 | Liao, YM 2020 | Swine | Central | Blood, Serum | ELISA | 3 | High | 1138 | 180 |
| 65 | Liu, F 2019 | Cattle | Northeastern | Blood, Serum | IHA | 3 | High | 535 | 33 |
| 66 | Liu, HL 2014 | Swine | Eastern | Blood, Serum | IHA | 3 | High | 379 | 78 |
| 67 | Liu, K 2015 | Swine | Central | Blood, Serum | ELISA | 3 | High | 1462 | 339 |
| 68 | Liu, LJ 2015 | Swine | Southwestern | Blood, Serum | ELISA | 2 | Moderate | 350 | 37 |
| 69 | Liu, LY 2016 | Swine | Northwestern | Blood, Serum | ELISA | 2 | Moderate | 173 | 143 |
| 70 | Liu, LY 2019 | Sheep | Northwestern | Blood, Serum | ELISA | 3 | High | 200 | 5 |
|  |  | Sheep |  |  | IHA |  |  | 203 | 9 |
| 71 | Liu, JW 2010 | Swine | Central | Blood, Serum | IHA | 2 | Moderate | 50 | 7 |
| 72 | Liu, Q 2010 | Sheep | Northwestern | Blood, Serum | IHA | 3 | High | 580 | 173 |
| 73 | Liu, Q 2011 | Cattle | Northwestern | Blood, Serum | IHA | 3 | High | 650 | 228 |
| 74 | Liu, SY 2017 | Chicken | Northeastern | Blood, Serum | IHA | 3 | High | 500 | 54 |
| 75 | Liu, X 2012 | Cattle | Northeastern | Blood, Serum | IHA | 3 | High | 646 | 39 |
|  |  | Swine |  |  |  |  |  | 1164 | 140 |
| 76 | Liu, X 2015 | Goat | Northwestern | Blood, Serum | IHA | 3 | High | 332 | 99 |
| 77 | Liu, X 2022 | Swine | Eastern | Blood, Serum | ELISA | 3 | High | 1170 | 244 |
| 78 | Liu, XC 2017 | Chicken | Eastern | Blood, Serum | ELISA | 3 | High | 350 | 235 |
| 79 | Liu, YM 2021 | Goat | Northeastern | Blood, Serum | ELISA | 4 | High | 265 | 19 |
|  |  | Goat | Eastern |  |  |  |  | 364 | 47 |
| 80 | Liu, YM 2022 | Cattle | Northeastern | Blood, Serum | ELISA | 4 | High | 894 | 64 |
| 81 | Liu, ZK 2015 | Goat | Northwestern | Blood, Serum | ELISA | 4 | High | 650 | 192 |
|  |  | Sheep |  |  |  |  |  | 600 | 128 |
| 82 | Luo, HQ 2016 | Goat | Central | Blood, Serum | ELISA | 3 | High | 2007 | 269 |
| 83 | Lu, Y 2012 | Cattle | Northwestern | Blood, Serum | ELISA | 3 | High | 898 | 21 |
| 84 | Luo, HQ 2017 | Goat | Eastern | Blood, Serum | IHA | 3 | High | 340 | 35 |
| 85 | Lv, JJ 2014 | Cattle | Northwestern | Blood, Serum | IHA | 2 | Moderate | 84 | 22 |
| 86 | Lv, QY 2021 | Chicken | Central | Blood, Serum | IHA | 3 | High | 1360 | 457 |
| 87 | Ma, L 2015 | Chicken | Northeastern | Blood, Serum | ELISA | 3 | High | 1095 | 193 |
| 88 | Ma, L 2021 | Cattle | Northern | Blood, Serum | ELISA | 3 | High | 723 | 144 |
| 89 | Ma, SX 2020 | Goat | Eastern | Blood, Serum | ELISA | 3 | High | 642 | 68 |
| 90 | Mao, KM 2013 | Goat | Eastern | Blood, Serum | IHA | 3 | High | 210 | 43 |
| 91 | Meng, R 2018 | Cattle | Central | Blood, Serum | IHA | 3 | High | 324 | 24 |
| 92 | OuYang, X 2014 | Goat | Southwestern | Blood, Serum | IHA | 3 | High | 427 | 27 |
| 93 | Pan, YY 2019 | Swine | Central | Blood, Serum | ELISA | 3 | High | 742 | 267 |
| 94 | Qin, SY 2015 | Cattle | Northwestern | Blood, Serum | MAT | 3 | High | 974 | 155 |
| 95 | Qiu, JH 2012 | Cattle | Northeast | Blood, Serum | IHA | 4 | High | 1803 | 46 |
| 96 | Qiu, MZ 2018 | Swine | Central | Blood, Serum | ELISA | 1 | Low | 184 | 108 |
| 97 | Ren, QJ 2011 | Cattle | Northwestern | Blood, Serum | IHA | 3 | High | 360 | 21 |
| 98 | Ren, XR 2012 | Cattle | Northwestern | Blood, Serum | IHA | 2 | Moderate | 120 | 17 |
| 99 | Ren, XY 2017 | Swine | Eastern | Blood, Serum | IHA | 3 | High | 317 | 11 |
| 100 | Shao, JL 2017 | Swine | Eastern | Blood, Serum | IHA | 3 | High | 478 | 192 |
| 101 | Su, R 2020 | Swine | Central | Blood, Serum | MAT | 3 | High | 407 | 95 |
| 102 | Sun, CX 2018 | Swine | Southwestern | Blood, Serum | ELISA | 3 | High | 631 | 14 |
| 103 | Sun, HY 2016 (a) | Swine | Central | Blood, Serum | ELISA | 3 | High | 4680 | 1635 |
| 104 | Sun, HY 2016 (b) | Chicken | Central | Blood, Serum | IHA | 3 | High | 3774 | 536 |
| 105 | Sun, LX 2020 | Goat | Southwestern | Blood, Serum | MAT | 3 | High | 169 | 59 |
|  |  | Sheep |  |  |  |  |  | 312 | 118 |
| 106 | Sun, T 2021 | Cattle | Northwestern | Blood, Serum | ELISA | 4 | High | 1101 | 95 |
|  |  | Cattle | Southwestern |  |  |  |  | 1683 | 166 |
| 107 | Sun, WW 2015 | Cattle | Northeastern | Blood, Serum | IHA | 4 | High | 2005 | 213 |
|  |  | Cattle | Northern |  |  |  |  | 1669 | 177 |
|  |  | Cattle | Eastern |  |  |  |  | 813 | 80 |
| 108 | Tan, QD 2015 | Cattle | Northwestern | Blood, Serum | MAT | 3 | High | 1657 | 80 |
| 109 | Tang, WQ 2022 | Chicken | Central | Blood, Serum | IHA | 3 | High | 350 | 118 |
| 110 | Tang, WY 2020 | Cattle | Northwestern | Blood, Serum | IHA | 3 | High | 498 | 87 |
| 111 | Tang, XM 2018 | Goat | Central | Blood, Serum | IHA | 3 | High | 5600 | 468 |
| 112 | Tao, Q 2011 | Swine | Central | Blood, Serum | ELISA | 4 | High | 3558 | 873 |
| 113 | Tian, HR 2016 | Goat | Southwestern | Blood, Serum | ELISA | 2 | Moderate | 126 | 0 |
| 114 | Tian, PR 2010 | Chicken | Northern | Blood, Serum | IHA | 3 | High | 580 | 43 |
| 115 | Wang, D 2016 | Swine | Northeastern | Tissue | MAT | 2 | Moderate | 2063 | 233 |
| 116 | Wang, HB 2012 | Goat | Central | Blood, Serum | ELISA | 3 | High | 966 | 0 |
| 117 | Wang, J 2013 | Cattle | Central | Blood, Serum | ELISA | 3 | High | 800 | 43 |
| 118 | Wang, L 2020 | Swine | Southern | Blood, Serum | ELISA | 3 | High | 95 | 1 |
|  |  | Swine | Southwestern |  |  |  |  | 76 | 0 |
|  |  | Swine | Eastern |  |  |  |  | 83 | 4 |
| 119 | Wang, M 2011 | Goat | Northestern | Blood, Serum | IHA | 3 | High | 360 | 15 |
| 120 | Wang, M 2012 | Cattle | Northwestern | Blood, Serum | IHA | 3 | High | 1603 | 133 |
| 121 | Wang, M 2015 | Cattle | Northwestern | Blood, Serum | IHA | 3 | High | 397 | 96 |
| 122 | Wang, QJ 2018 | Sheep | Northwestern | Blood, Serum | IHA | 3 | High | 12505 | 13 |
|  |  | Cattle |  |  |  |  |  | 1439 | 151 |
| 123 | Wang, QQ 2016 | Sheep | Northwestern | Blood, Serum | IHA | 3 | High | 486 | 10 |
| 124 | Wang, SG 2019 | Cattle | Northwestern | Blood, Serum | IHA | 1 | Low | 103 | 0 |
| 125 | Wang, WZ 2012 | Cattle | Northwestern | Blood, Serum | IHA | 3 | High | 240 | 34 |
| 126 | Wang, YG 2019 | Swine | Central | Blood, Serum | IHA | 3 | High | 612 | 49 |
| 127 | Wang, YY 2018 | Swine | Eastern | Blood, Serum | ELISA | 4 | High | 321 | 189 |
| 128 | Wang, ZY 2018 | Cattle | Southwestern | Blood, Serum | ELISA | 4 | High | 345 | 94 |
| 129 | Wen, QN 2015 | Swine | Central | Blood, Serum | ELISA | 3 | High | 2642 | 627 |
| 130 | Wu, DY 2012 | Swine | Southwestern | Blood, Serum | IHA | 2 | Moderate | 908 | 278 |
| 131 | Wu, F 2017 | Swine | Northwestern | Blood, Serum | IHA | 3 | High | 784 | 156 |
| 132 | Wu, SJ 2018 | Chicken | Northeastern | Blood, Serum | ELISA | 3 | High | 339 | 66 |
| 133 | Wu, SM 2011 | Sheep | Southwestern | Blood, Serum | IHA | 3 | High | 455 | 26 |
| 134 | Wu, SM 2012 | Swine | Southwestern | Blood, Serum | MAT | 3 | High | 427 | 97 |
| 135 | Xiang, ZJ 2011 | Swine | Eastern | Blood, Serum | IHA | 2 | Moderate | 1227 | 298 |
| 136 | Xie, WT 2019 | Swine | Central | Blood, Serum | ELISA | 3 | High | 4203 | 1627 |
| 137 | Xing, DY 2022 | Sheep | Northwestern | Blood, Serum | IHA | 3 | High | 272 | 6 |
| 138 | Xu, B 2013 | Swine | Southwestern | Blood, Serum | ELISA | 3 | High | 11700 | 8886 |
| 139 | Xu, MJ 2012 | Cattle | Southern | Blood, Serum | IHA | 3 | High | 875 | 120 |
| 140 | Xu, P 2012 | Chicken | Northeastern | Blood, Serum | MAT | 3 | High | 610 | 55 |
| 141 | Xu, P 2014 | Goat | Northeastern | Blood, Serum | IHA | 3 | High | 650 | 58 |
| 142 | Xu, P 2015 (a) | Goat | Northeastern | Blood, Serum | MAT | 4 | High | 216 | 32 |
|  |  | Sheep |  |  |  |  |  | 402 | 72 |
| 143 | Xu, P 2015 (b) | Swine | Northeastern | Blood, Serum | IHA | 3 | High | 1235 | 236 |
| 144 | Xu, Y 2014 | Swine | Central | Blood, Serum | IHA | 3 | High | 1191 | 373 |
| 145 | Xu, ZM 2016 | Swine | Northwestern | Blood, Serum | ELISA | 3 | High | 433 | 55 |
| 146 | Yan, P 2020 | Swine | Eastern | Blood, Serum | ELISA | 3 | High | 201 | 74 |
|  |  | Swine | Northern |  |  |  |  | 262 | 132 |
| 147 | Yan, XL 2020 | Sheep | Northern | Blood, Serum | ELISA | 4 | High | 1853 | 286 |
| 148 | Yan, XL 2021 | Sheep | Northern | Blood, Serum | ELISA | 4 | High | 641 | 140 |
| 149 | Yang, MY 2011 | Swine | Southwestern | Blood, Serum | IHA | 1 | Low | 80 | 14 |
| 150 | Yang, N 2012 | Chicken | Northeastern | Blood, Serum | MAT | 3 | High | 502 | 37 |
| 151 | Yang, N 2013 | Sheep | Northeastern | Blood, Serum | IHA | 3 | High | 566 | 25 |
| 152 | Yang, N 2017 | Swine | Northeastern | Blood, Serum | MAT | 2 | Moderate | 2063 | 233 |
| 153 | Yang, QF 2023 | Swine | Southwestern | Blood, Serum | IHA | 3 | High | 850 | 90 |
| 154 | Yang, XW 2015 | Swine | Southwestern | Blood, Serum | ELISA | 3 | High | 1109 | 100 |
| 155 | Yang, ZB 2017 | Swine | Southern | Blood, Serum | IHA | 3 | High | 30024 | 3364 |
| 156 | Yin, MY 2015 (a) | Cattle | Northwestern | Blood, Serum | MAT | 3 | High | 610 | 122 |
| 157 | Yin, MY 2015 (b) | Cattle | Northwestern | Blood, Serum | MAT | 4 | High | 1732 | 352 |
| 158 | Yin, YS 2015 | Swine | Southwestern | Blood, Serum | IHA | 3 | High | 925 | 175 |
| 159 | You, JZ 2015 | Swine | Eastern | Blood, Serum | IHA | 3 | High | 402 | 140 |
| 160 | Yu, BB 2018 | Cattle | Northeastern | Blood, Serum | ELISA | 3 | High | 318 | 34 |
| 161 | Yu, HJ 2011 | Swine | Eastern | Blood, Serum | ELISA | 3 | High | 813 | 434 |
| 162 | Zhang, BC 2018 | Swine | Eastern | Blood, Serum | IHA | 3 | High | 775 | 59 |
| 163 | Zhang, HB 2014 | Sheep | Northwestern | Blood, Serum | IHA | 1 | Low | 97 | 4 |
|  |  | Cattle |  |  |  |  |  | 105 | 3 |
| 164 | Zhang, N 2013 | Sheep | Central | Blood, Serum | MAT | 4 | High | 779 | 92 |
| 165 | Zhang, XQ 2010 | Cattle | Northwestern | Blood, Serum | IHA | 3 | High | 214 | 13 |
| 166 | Zhang, Y 2020 | Swine | Eastern | Blood, Serum | ELISA | 4 | High | 1158 | 160 |
| 167 | Zhang, Y 2022 | Swine | Southwestern | Blood, Serum | ELISA | 4 | High | 1221 | 136 |
| 168 | Zhao, G 2012 | Chicken | Eastern | Blood, Serum | ELISA | 3 | High | 360 | 122 |
|  |  | Chicken | Southern |  |  |  |  | 212 | 73 |
|  |  | Chicken | Central |  |  |  |  | 246 | 71 |
|  |  | Chicken | Northern |  |  |  |  | 61 | 15 |
|  |  | Chicken | Northwestern |  |  |  |  | 67 | 11 |
|  |  | Chicken | Northeastern |  |  |  |  | 50 | 10 |
|  |  | Chicken | Southwestern |  |  |  |  | 177 | 54 |
| 169 | Zhao, GH 2011 | Goat | Northwestern | Blood, Serum | IHA | 4 | High | 751 | 106 |
| 170 | Zhao, L 2015 (a) | Cattle | Northwestern | Blood, Serum | IHA | 3 | High | 200 | 13 |
| 171 | Zhao, L 2015 (b) | Sheep | Northwestern | Blood, Serum | IHA | 3 | High | 200 | 12 |
| 172 | Zhao, P 2016 | Cattle | Northeastern | Blood, Serum | ELISA | 2 | Moderate | 201 | 12 |
| 173 | Zhao, QB 2011 | Sheep | Northwestern | Blood, Serum | IHA | 2 | Moderate | 32 | 10 |
|  |  | Goat |  |  |  |  |  | 178 | 36 |
| 174 | Zhao, ZG 2018 | Cattle | Northwestern | Blood, Serum | IHA | 2 | Moderate | 300 | 2 |
| 175 | Zheng, B 2017 (a) | Chicken | Central | Blood, Serum | IHA | 3 | High | 551 | 31 |
| 176 | Zheng, B 2017 (b) | Swine | Central | Blood, Serum | IHA | 3 | High | 428 | 109 |
| 177 | Zheng, LG 2019 | Goat | Southwestern | Blood, Serum | ELISA | 4 | High | 356 | 171 |
| 178 | Zhou, DH 2010 | Swine | Southern | Blood, Serum | ELISA | 3 | High | 1022 | 276 |
| 179 | Zhou, DH 2012 | Cattle | Southern | Blood, Serum | IHA | 3 | High | 350 | 20 |
| 180 | Zhou, XH 2018 | Goat | Central | Blood, Serum | ELISA | 3 | High | 132 | 25 |
|  |  | Sheep |  |  |  |  |  | 502 | 78 |
| 181 | Zhou, XX 2014 | Cattle | Northern | Blood, Serum | IHA | 3 | High | 49 | 0 |
|  |  | Cattle | Central |  |  |  |  | 102 | 0 |
|  |  | Sheep | Multi-region |  |  |  |  | 208 | 10 |
|  |  | Goat | Multi-region |  |  |  |  | 243 | 16 |
| 182 | Zhou, YT 2022 | Swine | Northeastern | Blood, Serum | ELISA | 3 | High | 326 | 92 |
| 183 | Zhou, Z 2018 | Goat | Southwestern | Blood, Serum | ELISA | 3 | High | 332 | 141 |
| 184 | Zou, F 2015 | Sheep | Southwestern | Blood, Serum | IHA | 3 | High | 154 | 15 |
|  |  | Goat |  |  |  |  |  | 392 | 69 |

^#^ Cattle, Dairy cows, and Yaks are collectively classified as 'cattle'
