# Supplementary material for: Deciphering the epidemiological dynamics: Toxoplasma gondii seroprevalence in mainland China’s food animals, 2010-2023
Source: Front Cell Infect Microbiol. 2024 Apr 3;14:1381537. doi: 10.3389/fcimb.2024.1381537 (PMC11021580; doi:10.3389/fcimb.2024.1381537)
Supplement: Supplementary file 3 [file Table_3.docx]

**Table S6. Quality assessment of included studies.**

| ID | Study | Detection method | Sample size≥200 | Sample-collection method | Four or more risk factors | Quality score |
| --- | --- | --- | --- | --- | --- | --- |
| 1 | Bai, MJ 2017 | 1 | 1 | 1 | 1 | 4 |
| 2 | Bai, PX 2022 | 1 | 1 | 1 | 0 | 3 |
| 3 | Cai, GJ 2019 | 1 | 1 | 1 | 0 | 3 |
| 4 | Cai, JZ 2011 | 1 | 1 | 1 | 0 | 3 |
| 5 | Cai, WM 2018 | 1 | 1 | 1 | 0 | 3 |
| 6 | Cai, ZC 2017 | 1 | 1 | 0 | 0 | 2 |
| 7 | Chang, QC 2013 | 1 | 1 | 1 | 0 | 3 |
| 8 | Chen, CL 2012 | 1 | 1 | 1 | 0 | 3 |
| 9 | Chen, CJ 2017 | 1 | 1 | 1 | 0 | 3 |
| 10 | Chen, QL 2014 | 1 | 1 | 1 | 0 | 3 |
| 11 | Chen, WC 2022 | 1 | 1 | 1 | 1 | 4 |
| 12 | Chen, Y 2017 | 1 | 1 | 1 | 0 | 3 |
| 13 | Chen, YJ 2010 | 1 | 1 | 1 | 0 | 3 |
| 14 | Cong, W 2012 | 1 | 1 | 1 | 0 | 3 |
| 15 | Cui, P 2010 | 1 | 1 | 1 | 0 | 3 |
| 16 | Dai, D 2014 | 1 | 1 | 1 | 0 | 3 |
| 17 | Deng, ZH 2010 | 1 | 1 | 0 | 0 | 2 |
| 18 | Dong, BY 2014 | 1 | 1 | 1 | 0 | 3 |
| 19 | Dong, H 2018 | 1 | 1 | 1 | 0 | 3 |
| 20 | Dong, YS 2010 | 1 | 1 | 1 | 0 | 3 |
| 21 | Dong, YS 2011 | 1 | 1 | 1 | 0 | 3 |
| 22 | Feng, Y 2022 | 1 | 1 | 1 | 0 | 3 |
| 23 | Feng, YJ 2016 (a) | 1 | 1 | 0 | 0 | 2 |
| 24 | Feng, YJ 2016 (b) | 1 | 1 | 1 | 0 | 3 |
| 25 | Gao, WX 2016 | 1 | 1 | 1 | 0 | 3 |
| 26 | Gao, Y 2018 | 1 | 1 | 1 | 1 | 4 |
| 27 | Ge, W 2014 | 1 | 1 | 1 | 0 | 3 |
| 28 | Gu, DH 2014 | 1 | 1 | 1 | 0 | 3 |
| 29 | Han, HL 2019 | 1 | 1 | 1 | 0 | 3 |
| 30 | Han, JQ 2011 | 1 | 1 | 1 | 0 | 3 |
| 31 | He, JG 2015 | 1 | 1 | 1 | 0 | 3 |
| 32 | Hong, NN 2010 | 1 | 1 | 1 | 0 | 3 |
| 33 | Hu, KC 2020 | 1 | 1 | 1 | 0 | 3 |
| 34 | Hu, XH 2023 | 1 | 1 | 1 | 0 | 3 |
| 35 | Huang, CQ 2010 | 1 | 1 | 1 | 0 | 3 |
| 36 | Jia, T 2023 | 1 | 1 | 1 | 0 | 3 |
| 37 | Jiang, FC 2013 | 1 | 1 | 1 | 0 | 3 |
| 38 | Jiang, HH 2014 | 1 | 1 | 1 | 0 | 3 |
| 39 | Jiang, T 2010 | 1 | 1 | 1 | 0 | 3 |
| 40 | Jiang, Y 2011 | 1 | 1 | 0 | 0 | 2 |
| 41 | Jiang, Y 2023 | 1 | 1 | 0 | 0 | 2 |
| 42 | Jiang, YH 2015 | 1 | 1 | 1 | 0 | 3 |
| 43 | Jinan, CR 2015 | 1 | 1 | 1 | 0 | 3 |
| 44 | Kang, M 2013 | 1 | 0 | 1 | 0 | 2 |
| 45 | Lei, CH 2014 | 1 | 1 | 1 | 0 | 3 |
| 46 | Lei, CH 2015 | 1 | 0 | 1 | 0 | 2 |
| 47 | Li, CY 2018 | 1 | 1 | 1 | 0 | 3 |
| 48 | Li, F 2016 | 1 | 1 | 1 | 1 | 4 |
| 49 | Li, G 2021 | 1 | 1 | 1 | 0 | 3 |
| 50 | Li, JH 2021 | 1 | 1 | 0 | 0 | 2 |
| 51 | Li, JN 2018 | 1 | 1 | 1 | 1 | 4 |
| 52 | Li, K 2014 | 1 | 1 | 1 | 0 | 3 |
| 53 | Li, MH 2020 | 1 | 1 | 1 | 1 | 4 |
| 54 | Li, Q 2019 | 1 | 1 | 1 | 1 | 4 |
| 55 | Li, QS 2022 | 1 | 1 | 1 | 0 | 3 |
| 56 | Li, W 2018 | 1 | 1 | 1 | 0 | 3 |
| 57 | Li, XP 2016 | 1 | 0 | 1 | 0 | 2 |
| 58 | Li, XP 2017 | 1 | 0 | 1 | 0 | 2 |
| 59 | Li, Y 2010 (a) | 1 | 0 | 1 | 0 | 2 |
| 60 | Li, Y 2010 (b) | 1 | 0 | 1 | 0 | 2 |
| 61 | Li, YG 2011 | 1 | 1 | 1 | 0 | 3 |
| 62 | Li, YN 2015 | 1 | 0 | 0 | 0 | 1 |
| 63 | Liao, GY 2016 | 1 | 1 | 1 | 1 | 4 |
| 64 | Liao, YM 2020 | 1 | 1 | 0 | 1 | 3 |
| 65 | Liu, F 2019 | 1 | 1 | 1 | 0 | 3 |
| 66 | Liu, HL 2014 | 1 | 1 | 1 | 0 | 3 |
| 67 | Liu, K 2015 | 1 | 1 | 1 | 0 | 3 |
| 68 | Liu, LJ 2015 | 1 | 1 | 0 | 0 | 2 |
| 69 | Liu, LY 2016 | 1 | 0 | 1 | 0 | 2 |
| 70 | Liu, LY 2019 | 1 | 1 | 1 | 0 | 3 |
| 71 | Liu, JW 2010 | 1 | 0 | 1 | 0 | 2 |
| 72 | Liu, Q 2010 | 1 | 1 | 1 | 0 | 3 |
| 73 | Liu, Q 2011 | 1 | 1 | 1 | 0 | 3 |
| 74 | Liu, SY 2017 | 1 | 1 | 1 | 0 | 3 |
| 75 | Liu, X 2012 | 1 | 1 | 1 | 0 | 3 |
| 76 | Liu, X 2015 | 1 | 1 | 1 | 0 | 3 |
| 77 | Liu, X 2022 | 1 | 1 | 1 | 0 | 3 |
| 78 | Liu, XC 2017 | 1 | 1 | 1 | 0 | 3 |
| 79 | Liu, YM 2021 | 1 | 1 | 1 | 1 | 4 |
| 80 | Liu, YM 2022 | 1 | 1 | 1 | 1 | 4 |
| 81 | Liu, ZK 2015 | 1 | 1 | 1 | 1 | 4 |
| 82 | Luo, HQ 2016 | 1 | 1 | 1 | 0 | 3 |
| 83 | Lu, Y 2012 | 1 | 1 | 1 | 0 | 3 |
| 84 | Luo, HQ 2017 | 1 | 1 | 1 | 0 | 3 |
| 85 | Lv, JJ 2014 | 1 | 0 | 1 | 0 | 2 |
| 86 | Lv, QY 2021 | 1 | 1 | 1 | 0 | 3 |
| 87 | Ma, L 2015 | 1 | 1 | 1 | 0 | 3 |
| 88 | Ma, L 2021 | 1 | 1 | 1 | 0 | 3 |
| 89 | Ma, SX 2020 | 1 | 1 | 1 | 0 | 3 |
| 90 | Mao, KM 2013 | 1 | 1 | 1 | 0 | 3 |
| 91 | Meng, R 2018 | 1 | 1 | 1 | 0 | 3 |
| 92 | OuYang, X 2014 | 1 | 1 | 1 | 0 | 3 |
| 93 | Pan, YY 2019 | 1 | 1 | 1 | 0 | 3 |
| 94 | Qin, SY 2015 | 1 | 1 | 0 | 1 | 3 |
| 95 | Qiu, JH 2012 | 1 | 1 | 1 | 1 | 4 |
| 96 | Qiu, MZ 2018 | 1 | 0 | 0 | 0 | 1 |
| 97 | Ren, QJ 2011 | 1 | 1 | 1 | 0 | 3 |
| 98 | Ren, XR 2012 | 1 | 0 | 1 | 0 | 2 |
| 99 | Ren, XY 2017 | 1 | 1 | 1 | 0 | 3 |
| 100 | Shao, JL 2017 | 1 | 1 | 1 | 0 | 3 |
| 101 | Su, R 2020 | 1 | 1 | 1 | 0 | 3 |
| 102 | Sun, CX 2018 | 1 | 1 | 1 | 0 | 3 |
| 103 | Sun, HY 2016 (a) | 1 | 1 | 1 | 0 | 3 |
| 104 | Sun, HY 2016 (b) | 1 | 1 | 1 | 0 | 3 |
| 105 | Sun, LX 2020 | 1 | 1 | 1 | 0 | 3 |
| 106 | Sun, T 2021 | 1 | 1 | 1 | 1 | 4 |
| 107 | Sun, WW 2015 | 1 | 1 | 1 | 1 | 4 |
| 108 | Tan, QD 2015 | 1 | 1 | 1 | 0 | 3 |
| 109 | Tang, WQ 2022 | 1 | 1 | 1 | 0 | 3 |
| 110 | Tang, WY 2020 | 1 | 1 | 1 | 0 | 3 |
| 111 | Tang, XM 2018 | 1 | 1 | 1 | 0 | 3 |
| 112 | Tao, Q 2011 | 1 | 1 | 1 | 1 | 4 |
| 113 | Tian, HR 2016 | 1 | 0 | 1 | 0 | 2 |
| 114 | Tian, PR 2010 | 1 | 1 | 1 | 0 | 3 |
| 115 | Wang, D 2016 | 1 | 1 | 0 | 0 | 2 |
| 116 | Wang, HB 2012 | 1 | 1 | 1 | 0 | 3 |
| 117 | Wang, J 2013 | 1 | 1 | 1 | 0 | 3 |
| 118 | Wang, L 2020 | 1 | 1 | 1 | 0 | 3 |
| 119 | Wang, M 2011 | 1 | 1 | 1 | 0 | 3 |
| 120 | Wang, M 2012 | 1 | 1 | 1 | 0 | 3 |
| 121 | Wang, M 2015 | 1 | 1 | 1 | 0 | 3 |
| 122 | Wang, QJ 2018 | 1 | 1 | 1 | 0 | 3 |
| 123 | Wang, QQ 2016 | 1 | 1 | 1 | 0 | 3 |
| 124 | Wang, SG 2019 | 1 | 0 | 0 | 0 | 1 |
| 125 | Wang, WZ 2012 | 1 | 1 | 1 | 0 | 3 |
| 126 | Wang, YG 2019 | 1 | 1 | 1 | 0 | 3 |
| 127 | Wang, YY 2018 | 1 | 1 | 1 | 1 | 4 |
| 128 | Wang, ZY 2018 | 1 | 1 | 1 | 1 | 4 |
| 129 | Wen, QN 2015 | 1 | 1 | 1 | 0 | 3 |
| 130 | Wu, DY 2012 | 1 | 1 | 0 | 0 | 2 |
| 131 | Wu, F 2017 | 1 | 1 | 1 | 0 | 3 |
| 132 | Wu, SJ 2018 | 1 | 1 | 1 | 0 | 3 |
| 133 | Wu, SM 2011 | 1 | 1 | 1 | 0 | 3 |
| 134 | Wu, SM 2012 | 1 | 1 | 1 | 0 | 3 |
| 135 | Xiang, ZJ 2011 | 1 | 1 | 0 | 0 | 2 |
| 136 | Xie, WT 2019 | 1 | 1 | 0 | 1 | 3 |
| 137 | Xing, DY 2022 | 1 | 1 | 1 | 0 | 3 |
| 138 | Xu, B 2013 | 1 | 1 | 1 | 0 | 3 |
| 139 | Xu, MJ 2012 | 1 | 1 | 1 | 0 | 3 |
| 140 | Xu, P 2012 | 1 | 1 | 1 | 0 | 3 |
| 141 | Xu, P 2014 | 1 | 1 | 1 | 0 | 3 |
| 142 | Xu, P 2015 (a) | 1 | 1 | 1 | 1 | 4 |
| 143 | Xu, P 2015 (b) | 1 | 1 | 1 | 0 | 3 |
| 144 | Xu, Y 2014 | 1 | 1 | 1 | 0 | 3 |
| 145 | Xu, ZM 2016 | 1 | 1 | 1 | 0 | 3 |
| 146 | Yan, P 2020 | 1 | 1 | 1 | 0 | 3 |
| 147 | Yan, XL 2020 | 1 | 1 | 1 | 1 | 4 |
| 148 | Yan, XL 2021 | 1 | 1 | 1 | 1 | 4 |
| 149 | Yang, MY 2011 | 1 | 0 | 0 | 0 | 1 |
| 150 | Yang, N 2012 | 1 | 1 | 1 | 0 | 3 |
| 151 | Yang, N 2013 | 1 | 1 | 1 | 0 | 3 |
| 152 | Yang, N 2017 | 1 | 1 | 0 | 0 | 2 |
| 153 | Yang, QF 2023 | 1 | 1 | 0 | 1 | 3 |
| 154 | Yang, XW 2015 | 1 | 1 | 1 | 0 | 3 |
| 155 | Yang, ZB 2017 | 1 | 1 | 1 | 0 | 3 |
| 156 | Yin, MY 2015 (a) | 1 | 1 | 1 | 0 | 3 |
| 157 | Yin, MY 2015 (b) | 1 | 1 | 1 | 1 | 4 |
| 158 | Yin, YS 2015 | 1 | 1 | 1 | 0 | 3 |
| 159 | You, JZ 2015 | 1 | 1 | 1 | 0 | 3 |
| 160 | Yu, BB 2018 | 1 | 1 | 1 | 0 | 3 |
| 161 | Yu, HJ 2011 | 1 | 1 | 1 | 0 | 3 |
| 162 | Zhang, BC 2018 | 1 | 1 | 1 | 0 | 3 |
| 163 | Zhang, HB 2014 | 1 | 0 | 0 | 0 | 1 |
| 164 | Zhang, N 2013 | 1 | 1 | 1 | 1 | 4 |
| 165 | Zhang, XQ 2010 | 1 | 1 | 1 | 0 | 3 |
| 166 | Zhang, Y 2020 | 1 | 1 | 1 | 1 | 4 |
| 167 | Zhang, Y 2022 | 1 | 1 | 1 | 1 | 4 |
| 168 | Zhao, G 2012 | 1 | 1 | 1 | 0 | 3 |
| 169 | Zhao, GH 2011 | 1 | 1 | 1 | 1 | 4 |
| 170 | Zhao, L 2015 (a) | 1 | 1 | 1 | 0 | 3 |
| 171 | Zhao, L 2015 (b) | 1 | 1 | 1 | 0 | 3 |
| 172 | Zhao, P 2016 | 1 | 0 | 1 | 0 | 2 |
| 173 | Zhao, QB 2011 | 1 | 0 | 1 | 0 | 2 |
| 174 | Zhao, ZG 2018 | 1 | 1 | 0 | 0 | 2 |
| 175 | Zheng, B 2017 (a) | 1 | 1 | 1 | 0 | 3 |
| 176 | Zheng, B 2017 (b) | 1 | 1 | 1 | 0 | 3 |
| 177 | Zheng, LG 2019 | 1 | 1 | 1 | 1 | 4 |
| 178 | Zhou, DH 2010 | 1 | 1 | 1 | 0 | 3 |
| 179 | Zhou, DH 2012 | 1 | 1 | 1 | 0 | 3 |
| 180 | Zhou, XH 2018 | 1 | 1 | 1 | 0 | 3 |
| 181 | Zhou, XX 2014 | 1 | 1 | 1 | 0 | 3 |
| 182 | Zhou, YT 2022 | 1 | 1 | 1 | 0 | 3 |
| 183 | Zhou, Z 2018 | 1 | 1 | 1 | 0 | 3 |
| 184 | Zou, F 2015 | 1 | 0 | 1 | 1 | 3 |
