# Supplementary material for: Deciphering the epidemiological dynamics: Toxoplasma gondii seroprevalence in mainland China’s food animals, 2010-2023
Source: Front Cell Infect Microbiol. 2024 Apr 3;14:1381537. doi: 10.3389/fcimb.2024.1381537 (PMC11021580; doi:10.3389/fcimb.2024.1381537)
Supplement: Supplementary file 4 [file Table_4.docx]

**Table S7. Pooled prevalence of *T. gondii* across different regions in 2010-2017 and 2018-2023.**

|  | **2010-2017** | | | | **2018-2023** | | | |  | |
| --- | --- | --- | --- | --- | --- | --- | --- | --- | --- | --- |
|  | **No. of Study** | **Positive** | **n** | **Prevalence (95% CI)** | **No. of Study** | **Positive** | **n** | **Prevalence** | **Z** | ***P* value** |
| Overall | 147 | 143695 | 31295 | 15.2% (12.5, 18.1) | 74 | 68290 | 9515 | 15.6% (11.9, 19.7) | 0.163 | 0.87 |
| Southwestern | 21 | 26118 | 13267 | 25.5% (13.8, 39.3) | 14 | 7873 | 1218 | 16.7% (9.3, 25.6) | -1.140 | 0.254 |
| Northern | 6 | 3498 | 450 | 10.7% (4.5, 19.0) | 6 | 5441 | 1183 | 25.2% (18.0, 33.1) | 2.715 | 0.006 |
| Southern and Central | 26 | 59556 | 9571 | 15.3% (11.7, 19.3) | 19 | 23831 | 3928 | 16.1% (9.6, 23.9) | 0.194 | 0.846 |
| Northwestern | 54 | 25620 | 3701 | 12.5% (9.9, 15.4) | 17 | 22083 | 1753 | 12.1% (4.1, 23.4) | -0.078 | 0.938 |
| Eastern | 17 | 9385 | 2309 | 23.4% (16.3, 31.3) | 9 | 4914 | 845 | 15.4% (7.8, 25.0) | 1.374 | 0.169 |
| Northeastern | 21 | 19067 | 1971 | 9.8% (7.7, 12.1) | 8 | 3860 | 501 | 13.6% (8.8, 19.3) | 0.922 | 0.356 |
| Mult-region | 2 | 451 | 26 | 5.7% (3.7, 8.1) | 1 | 288 | 87 | 30.2% (25.0, 35.9) | 8.170 | <0.001 |
