# Supplementary material for: Deciphering the epidemiological dynamics: Toxoplasma gondii seroprevalence in mainland China’s food animals, 2010-2023
Source: Front Cell Infect Microbiol. 2024 Apr 3;14:1381537. doi: 10.3389/fcimb.2024.1381537 (PMC11021580; doi:10.3389/fcimb.2024.1381537)
Supplement: Supplementary file 5 [file Table_5.docx]

**This study included 184 articles, published from 2010 to 2023** (Chen et al., 2010; Cui et al., 2010; Deng et al., 2010; Dong et al., 2010; Hong et al., 2010; Huang et al., 2010; Jiang et al., 2010; Li et al., 2010a; Li et al., 2010b; Liu et al., 2010a; Liu et al., 2010b; Tian and Cui, 2010; Zhang et al., 2010; Zhou et al., 2010; Cai and Li, 2011; Dong et al., 2011; Han et al., 2011; Jiang and Wu, 2011; Li, 2011; Liu et al., 2011; Ren et al., 2011; Tao et al., 2011; Wang, 2011; Wu et al., 2011; Xiang et al., 2011; Yang et al., 2011; Yu et al., 2011; Zhao et al., 2011a; Zhao et al., 2011b; Chen and Mao, 2012; Cong et al., 2012; Liu et al., 2012; Lu et al., 2012; Qiu et al., 2012; Ren, 2012; Wang et al., 2012a; Wang et al., 2012b; Wang, 2012; Wu et al., 2012a; Wu et al., 2012b; Xu et al., 2012a; Xu et al., 2012b; Yang et al., 2012; Zhao et al., 2012; Zhou et al., 2012; Chang et al., 2013; Jiang et al., 2013; Kang et al., 2013; Liu, 2013; Mao et al., 2013; Wang et al., 2013; Xu et al., 2013; Yang et al., 2013; Chen et al., 2014; Dai, 2014; Dong et al., 2014; Ge et al., 2014; Gu and Li, 2014; Jiang et al., 2014; Lei et al., 2014; Li et al., 2014; Liu, 2014; Lv et al., 2014; Ou et al., 2014; Xu et al., 2014a; Xu et al., 2014b; Zhang et al., 2014; Zhou et al., 2014; Jiang et al., 2015; Jin, 2015; Lei et al., 2015; Li et al., 2015; Liu et al., 2015a; Liu et al., 2015b; Liu et al., 2015c; M et al., 2015; Ma et al., 2015; Qin et al., 2015; Sun et al., 2015; Tan et al., 2015; Wang et al., 2015; Wen et al., 2015; Xu et al., 2015a; Xu et al., 2015b; Yang and Zhao, 2015; Yin et al., 2015a; Yin et al., 2015b; You et al., 2015; Zhao, 2015b; a; Zou et al., 2015; Feng et al., 2016a; Feng et al., 2016b; Gao et al., 2016; He and Wang, 2016; Li et al., 2016a; Li et al., 2016b; Liao et al., 2016; Liu et al., 2016; Luo et al., 2016; Sun, 2016; Sun et al., 2016; Tian et al., 2016; Wang et al., 2016a; Wang et al., 2016b; Xu et al., 2016; Zhang et al., 2016; Zhao et al., 2016; Bai et al., 2017; Cai et al., 2017; Chen et al., 2017a; Chen et al., 2017b; Li et al., 2017; Liu and Zhu, 2017; Liu et al., 2017; Luo et al., 2017; Shao, 2017; Wu et al., 2017; Yang et al., 2017a; Yang et al., 2017b; Zheng et al., 2017a; Zheng et al., 2017b; Cai et al., 2018; Dong et al., 2018; Gao et al., 2018; Li et al., 2018a; Li et al., 2018b; Li et al., 2018c; M et al., 2018; Meng, 2018; Sun et al., 2018; Tang et al., 2018; Wang et al., 2018a; Wang et al., 2018b; Wang et al., 2018c; Wu et al., 2018; Yu et al., 2018; Zhang et al., 2018; Zhao et al., 2018; Zhou, 2018; Zhou et al., 2018; Cai et al., 2019; Han, 2019; Li et al., 2019; Liu et al., 2019a; Liu et al., 2019b; Pan, 2019; Wang et al., 2019a; Wang et al., 2019b; Xie et al., 2019; Zheng and Feng, 2019; Hu, 2020; Li et al., 2020; Liao, 2020; Ma, 2020; Su et al., 2020; Sun et al., 2020; Tang et al., 2020; Wang et al., 2020; Yan et al., 2020a; Yan et al., 2020b; Zhang et al., 2020; Li et al., 2021; Li, 2021; Liu et al., 2021; Lv et al., 2021; Ma et al., 2021; Sun et al., 2021; Yan et al., 2021; Bai et al., 2022; Chen et al., 2022; Feng et al., 2022; Hu et al., 2022; Li, 2022; Liu et al., 2022a; Liu et al., 2022b; Ren, 2022; Tang et al., 2022; Xing et al., 2022; Zhang et al., 2022; Zhou et al., 2022; Jia et al., 2023; Jiang et al., 2023; Yang and Shi, 2023).

**67 articles were in English**(Huang et al., 2010; Liu et al., 2010b; Zhou et al., 2010; Liu et al., 2011; Tao et al., 2011; Wu et al., 2011; Yu et al., 2011; Zhao et al., 2011a; Cong et al., 2012; Liu et al., 2012; Qiu et al., 2012; Wang et al., 2012b; Wu et al., 2012a; Wu et al., 2012b; Xu et al., 2012a; Xu et al., 2012b; Yang et al., 2012; Zhao et al., 2012; Zhou et al., 2012; Chang et al., 2013; Yang et al., 2013; Ge et al., 2014; Jiang et al., 2014; Li et al., 2014; Xu et al., 2014a; Xu et al., 2014b; Li et al., 2015; Liu et al., 2015c; Ma et al., 2015; Qin et al., 2015; Sun et al., 2015; Tan et al., 2015; Xu et al., 2015a; Xu et al., 2015b; Yin et al., 2015a; Zou et al., 2015; Feng et al., 2016a; Li et al., 2016a; Luo et al., 2016; Wang et al., 2016a; Zhang et al., 2016; Bai et al., 2017; Liu et al., 2017; Luo et al., 2017; Wu et al., 2017; Dong et al., 2018; Gao et al., 2018; Zhou et al., 2018; Li et al., 2019; Liu et al., 2019a; Wang et al., 2019b; Li et al., 2020; Su et al., 2020; Sun et al., 2020; Yan et al., 2020b; Zhang et al., 2020; Li et al., 2021; Liu et al., 2021; Lv et al., 2021; Ma et al., 2021; Sun et al., 2021; Hu et al., 2022; Liu et al., 2022a; Liu et al., 2022b; Zhang et al., 2022; Jia et al., 2023; Jiang et al., 2023), **while 117 in Chinese** (Chen et al., 2010; Cui et al., 2010; Deng et al., 2010; Dong et al., 2010; Hong et al., 2010; Jiang et al., 2010; Li et al., 2010a; Li et al., 2010b; Liu et al., 2010a; Tian and Cui, 2010; Zhang et al., 2010; Cai and Li, 2011; Dong et al., 2011; Han et al., 2011; Jiang and Wu, 2011; Li, 2011; Ren et al., 2011; Wang, 2011; Xiang et al., 2011; Yang et al., 2011; Zhao et al., 2011b; Chen and Mao, 2012; Lu et al., 2012; Ren, 2012; Wang et al., 2012a; Wang, 2012; Jiang et al., 2013; Kang et al., 2013; Liu, 2013; Mao et al., 2013; Wang et al., 2013; Xu et al., 2013; Chen et al., 2014; Dai, 2014; Dong et al., 2014; Gu and Li, 2014; Lei et al., 2014; Liu, 2014; Lv et al., 2014; Ou et al., 2014; Zhang et al., 2014; Zhou et al., 2014; Jiang et al., 2015; Jin, 2015; Lei et al., 2015; Liu et al., 2015a; Liu et al., 2015b; M et al., 2015; Wang et al., 2015; Wen et al., 2015; Yang and Zhao, 2015; Yin et al., 2015b; You et al., 2015; Zhao, 2015b; a; Feng et al., 2016b; Gao et al., 2016; He and Wang, 2016; Li et al., 2016b; Liao et al., 2016; Liu et al., 2016; Sun, 2016; Sun et al., 2016; Tian et al., 2016; Wang et al., 2016b; Xu et al., 2016; Zhao et al., 2016; Cai et al., 2017; Chen et al., 2017a; Chen et al., 2017b; Li et al., 2017; Liu and Zhu, 2017; Shao, 2017; Yang et al., 2017a; Yang et al., 2017b; Zheng et al., 2017a; Zheng et al., 2017b; Cai et al., 2018; Li et al., 2018a; Li et al., 2018b; Li et al., 2018c; M et al., 2018; Meng, 2018; Sun et al., 2018; Tang et al., 2018; Wang et al., 2018a; Wang et al., 2018b; Wang et al., 2018c; Wu et al., 2018; Yu et al., 2018; Zhang et al., 2018; Zhao et al., 2018; Zhou, 2018; Cai et al., 2019; Han, 2019; Liu et al., 2019b; Pan, 2019; Wang et al., 2019a; Xie et al., 2019; Zheng and Feng, 2019; Hu, 2020; Liao, 2020; Ma, 2020; Tang et al., 2020; Wang et al., 2020; Yan et al., 2020a; Li, 2021; Yan et al., 2021; Bai et al., 2022; Chen et al., 2022; Feng et al., 2022; Li, 2022; Ren, 2022; Tang et al., 2022; Xing et al., 2022; Zhou et al., 2022; Yang and Shi, 2023)

**Reference**

Bai, M.J., Zou, Y., Elsheikha, H.M., Ma, J.G., Zheng, W.B., Zhao, Q., et al. (2017). Toxoplasma gondii Infection in Farmed Wild Boars (Sus scrofa) in Three Cities of Northeast China. *Foodborne Pathog Dis* 14(7)**,** 379-385. doi: 10.1089/fpd.2016.2260.

Bai, P., Chen, C., Yu, M., Liu, Q., Xu, S., and Pan, X. (2022). Seroprevalence Study of Toxoplasma gondii in Large-Scale Dairy Farms in Xining Region. *The Chinese Livestock and Poultry Breeding* 18(6)**,** 5-6. doi: 10.3969/j.issn.1673-4556.2022.06.002.

Cai, G., Zhang, G., Wang, J., Wang, X., and Li, X. (2019). Serological investigation Investigation and Analysis of Pigs Toxoplasmosis in Hainan Region, Qinghai Province. *Journal of Domestic Animal Ecology* 40(3)**,** 69-71. doi: 10.3969/j.issn.1673-1182.2019.03.014.

Cai, J., and Li, C. (2011). The Serological Investigation and Epidemiological Analysis of Toxoplasmosis in Livestock in Qinghai Province. *Chinese Journal of Veterinary Medicine* 47(1)**,** 45-46. doi: 10.3969/j.issn.0529-6005.2011.01.020.

Cai, W., Gao, Y., Liu, D., Cheng, D., Xing, H., Yang, A., et al. (2018). Investigation of Gastrointestinal Parasites and Toxoplasma gondii Infection in Goats in Yangzhou Region. *Animal Husbandry & Veterinary Medicine* 50(7)**,** 108-112.

Cai, Z., Li, F., Hu, P., Guan, W., Li, C., and Cao, S. (2017). Serological Investigation and Analysis of Toxoplasma gondii in Selected Pig Farms in Suburban Areas of Beijing City. *Heilongjiang Animal Science and Veterinary Medicine* (4)**,** 103-104.

Chang, Q.C., Zheng, X., Qiu, J.H., Wang, C.R., and Zhu, X.Q. (2013). Seroprevalence of Toxoplasma gondii infection in fattening pigs in Northeast China. *J Parasitol* 99(3)**,** 544-545. doi: 10.1645/12-102.1.

Chen, C., Fan, X., Tan, M., and Liu, Q. (2017a). Serological Survey and Analysis of Brucellosis, Chlamydiosis, and Toxoplasmosis in Dairy Farms in Huangyuan County, Qinghai Province. *Chinese Qinghai Journal of Animal and Veterinary Sciences* 47(1)**,** 33-35.

Chen, C., and Mao, Y. (2012). Serological Investigation of Toxoplasmosis in Zunyi City. *GUIZHOU JOURNAL OF ANIMAL HUSBANDRY & VETERINARY MEDICINE* 36(5)**,** 32-33. doi: 10.3969/j.issn.1007-1474.2012.05.011.

Chen, Q., Ji, E., Wang, C., Tu, R., and Ba, Y. (2014). Survey Report on Toxoplasma gondii Infection in Mountainous Areas of Hejing County. *Contemporary Animal Husbandry* (5)**,** 18-19.

Chen, W., Chen, F., Fu, Y., Tang, C., and Xie, W. (2022). Epidemiological Investigation and Risk Factor Analysis of Toxoplasma gondii Infection in Yellow Cattle in Chenzhou City, Hunan Province. *China Animal Health Inspection* 39(5)**,** 12-17. doi: 10.3969/j.issn.1005-944X.2022.05.003.

Chen, Y., Huang, F., Shi, D., Song, Y., Wang, Y., Zhu, Z., et al. (2017b). Serological Investigation of Toxoplasmosis in Dairy Cattle in Some Areas of Henan Province. *Chinese Journal of Veterinary Medicine* 53(8)**,** 45-46.

Chen, Y., Wang, Q., Jiang, W., Liu, Y., Jing, Z., and Yan, Y. (2010). Study on the Prevalence of Toxoplasma gondii in Pigs in Shanghai. *Chinese Journal of Animal Infectious Diseases* 18(5)**,** 54-58. doi: 10.3969/j.issn.1674-6422.2010.05.011.

Cong, W., Huang, S.Y., Zhou, D.H., Xu, M.J., Wu, S.M., Yan, C., et al. (2012). First report of Toxoplasma gondii infection in market-sold adult chickens, ducks and pigeons in northwest China. *Parasit Vectors* 5**,** 110. doi: 10.1186/1756-3305-5-110.

Cui, P., Fang, S., Gu, X., Guo, B., and Sun, X. (2010). The Epidemiological Investigation of Chicken and Rabbit Toxoplasmosis in Zhangjiakou Bashang Area. *China Animal Health Inspection* (5)**,** 46-47. doi: 10.3969/j.issn.1005-944X.2010.05.024.

Dai, D. (2014). Survey of Toxoplasma gondii Infection in Pigs in Yushu Prefecture, Qinghai Province. *Modern Animal Husbandry* (8)**,** 53-53. doi: 10.3969/j.issn.1005-5959.2014.08.036.

Deng, Z., Yang, J., Sun, X., Xie, Y., Zhao, G., Duan, G., et al. (2010). Seroepidemiological Survey of Toxoplasmosis in Pigs. *China Animal Husbandry & Veterinary Medicine* 37(8)**,** 225-226.

Dong, B., Qian, D., Li, G., Wu, Y., Wang, X., Chen, B., et al. (2014). Seroepidemiological Survey of Caprine Toxoplasmosis in Qiannan Prefecture in goats. *Chinese Journal of Veterinary Medicine* 50(9)**,** 33-34. doi: 10.3969/j.issn.0529-6005.2014.09.011.

Dong, H., Lu, Y.Y., Su, R.J., Wang, Y.H., Wang, M.Y., Jiang, Y.B., et al. (2018). Low prevalence of antibodies against Toxoplasma gondii in dairy cattle from China's central region. *BMC Vet Res* 14(1)**,** 315. doi: 10.1186/s12917-018-1629-3.

Dong, Y., Luo, Z., Zhang, G., and Liu, X. (2011). Epidemiological Investigation of Bovine and Ovine Toxoplasmosis in Qinghai Province. *Chinese journal of Zoonoses* 27(4)**,** 359,363. doi: 10.3969/j.issn.1002-2694.2011.04.023.

Dong, Y., Pan, Y., Fan, X., Zhang, G., and Liu, X. (2010). Epidemiological Investigation of Toxoplasmosis in Cattle and Goats in Qinghai Province. *Heilongjiang Animal Science and Veterinary Medicine* (12)**,** 83-84.

Feng, Y., Cheng, W., Dong, X., J., Y., and Cheng, Z. (2022). Serological Investigation of Toxoplasmosis in Yunnan Semi-Fine Wool Sheep in Yongshan County, Yunnan. *Yunnan Journal of Animal Science and Veterinary Medicine* (2)**,** 21-22. doi: 10.3969/j.issn.1005-1341.2022.02.007.

Feng, Y., Lu, Y., Wang, Y., Liu, J., Zhang, L., and Yang, Y. (2016a). Toxoplasma gondii and Neospora caninum in Free-Range Chickens in Henan Province of China. *Biomed Res Int* 2016**,** 8290536. doi: 10.1155/2016/8290536.

Feng, Y., Wang, Y., and Yang, Y. (2016b). Epidemiological Serological Survey and Risk Factor Analysis of Ovine Neosporosis and Toxoplasmosis. *Chinese journal of Zoonoses* 32(7)**,** 613-617. doi: 10.3969/j.issn.1002-2694.2016.07.005.

Gao, W., Li, K., Han, Z., Wang, X., Wang, L., Zhang, H., et al. (2016). Serological Testing Report for Toxoplasma gondii in Tibetan Pigs in Certain Regions of Tibet. *Hubei Journal of Animal and Veterinary Sciences* 37(3)**,** 5-6. doi: 10.3969/j.issn.1007-273X.2016.03.002.

Gao, Y., Guo, H.P., Adjou Moumouni, P.F., Sun, M., Liu, M.M., Efstratiou, A., et al. (2018). Seroprevalence of Toxoplasma gondii infection in sheep from northern China. *Trop Biomed* 35(3)**,** 664-668.

Ge, W., Sun, H., Wang, Z., Xu, P., Wang, W., Mu, G., et al. (2014). Prevalence and genotype of Toxoplasma gondii infection in cattle from Jilin Province, northeastern China. *Vector Borne Zoonotic Dis* 14(6)**,** 399-402. doi: 10.1089/vbz.2013.1516.

Gu, D., and Li, H. (2014). Serological Testing for Toxoplasmosis in Pigs. Shandong Journal of Animal Science and Veterinary Medicine. *Shandong Journal of Animal Science and Veterinary Medicine* 35(7)**,** 53-54. doi: 10.3969/j.issn.1007-1733.2014.07.038.

Han, H. (2019). Serological Survey of Toxoplasmosis in Some Dairy Farms in Suiping County, Henan Province. *China Dairy* (5)**,** 50-51.

Han, J., Sun, X., Li, B., Duan, G., and Zhou, F. (2011). Serological Survey of Toxoplasmosis in Pigs in Yuxi City, Yunnan Province. *Heilongjiang Animal Science and Veterinary Medicine* (4)**,** 102-103.

He, J., and Wang, S. (2016). Seroepidemiological Survey of Toxoplasmosis in Yaks and Sheep in Tianzhu County, Gansu Province. *Animal Husbandry & Veterinary Medicine* 48(11)**,** 136-137.

Hong, N., Qian, D., Zhang, D., Hua, Y., Li, T., Ran, L., et al. (2010). Seroepidemiological Survey of Toxoplasmosis in Yaks and Sheep in Tianzhu County, Gansu Province. *Chinese Journal of Animal Infectious Diseases* 18(4)**,** 68-70. doi: 10.3969/j.issn.1674-6422.2010.04.013.

Hu, K. (2020). Serological Antibody Testing and Analysis of Toxoplasmosis in Pigs in Selected Areas of Pingdingshan City. *Rural Technology* (21)**,** 111-112. doi: 10.3969/j.issn.1674-7909.2020.21.056.

Hu, X.H., Xie, S.C., Liang, Q.L., Sun, L.X., Li, Z., Yang, J.F., et al. (2022). Seroprevalence and risk factors of Toxoplasma gondii and Neospora caninum infection in black goats in Yunnan Province, Southwestern China. *Front Vet Sci* 9**,** 975238. doi: 10.3389/fvets.2022.975238.

Huang, C.Q., Lin, Y.Y., Dai, A.L., Li, X.H., Yang, X.Y., Yuan, Z.G., et al. (2010). Seroprevalence of Toxoplasma gondii infection in breeding sows in Western Fujian Province, China. *Trop Anim Health Prod* 42(1)**,** 115-118. doi: 10.1007/s11250-009-9393-9.

Jia, T., Zhang, T.H., Yu, L.M., Wang, Z.R., Zheng, W.B., Liu, Q., et al. (2023). Seroprevalence of Toxoplasma gondii infection in sheep and cattle in Shanxi Province, North China. *Vet Parasitol Reg Stud Reports* 43**,** 100897. doi: 10.1016/j.vprsr.2023.100897.

Jiang, F., Jiang, S., Chen, S., and Sun, H. (2013). Serological Survey of Toxoplasmosis in Pigs in Huaian City. *Shanghai Journal of Animal Husbandry & Veterinary Medicine* (2)**,** 47,49. doi: 10.3969/j.issn.1000-7725.2013.02.024.

Jiang, H.H., Zhang, W.B., Zhao, L., Zhou, D.H., Song, H.Q., Xu, C.M., et al. (2014). Seroprevalence of Toxoplasma gondii infection in pigs in Jiangxi Province, Southeastern China. *Foodborne Pathog Dis* 11(5)**,** 362-365. doi: 10.1089/fpd.2013.1686.

Jiang, T., Yi, C., Ruan, Z., and He, H. (2010). Serological Investigation of Toxoplasma gondii in Chickens in Jingzhou City. *Poultry Husbandry and Disease Control* (2)**,** 38-39.

Jiang, Y., Wang, Z., Zhang, Y., Ye, Q., and Bo, X. (2015). Serological Survey of Toxoplasma gondii in Dairy Cattle in Certain Regions of Northern Xinjiang. *Chinese Journal of Veterinary Medicine* 51(9)**,** 56-57. doi: 10.3969/j.issn.0529-6005.2015.09.019.

Jiang, Y., and Wu, L. (2011). Seroepidemiological Survey of Toxoplasma gondii Infection in Selected Pig Farms in Xinluo District, Longyan City. *Fujian Journal of Animal Husbandry & Veterinary Medicine* 33(2)**,** 6-7. doi: 10.3969/j.issn.1003-4331.2011.02.003.

Jiang, Y., Xin, S., Ma, Y., Zhang, H., Yang, X., and Yang, Y. (2023). Low Prevalence of Toxoplasma gondii in Sheep and Isolation of a Viable Strain from Edible Mutton from Central China. *Pathogens* 12(6). doi: 10.3390/pathogens12060827.

Jin, A. (2015). Epidemiological Serological Survey of Toxoplasma gondii in Pigs in Chindu County, Qinghai Province. *Heilongjiang Animal Science and Veterinary Medicine* (8)**,** 110-111.

Kang, M., Li, Y., Shi, W., Ren, X., Zhang, C., and Zhang, L. (2013). Seroepidemiological Survey of Toxoplasmosis in Cattle and Sheep in Hualong County, Qinghai Province. *Animal Husbandry & Veterinary Medicine* 45(10)**,** 128.

Lei, C., Bian, S., Gao, D., Bao, Z., and Cai, Y. (2014). Seroepidemiological Survey of Toxoplasmosis in Sheep in the Altay Region of Xinjiang. *Anim Husb Feed Sci* 35(6)**,** 99-100. doi: 10.3969/j.issn.1672-5190.2014.06.049.

Lei, C., Cai, Y., Bao, Z., Bian, S., and Gao, D. (2015). Serological Survey of Toxoplasma gondii Infection Among Free-Range Chickens and Sparrows in Free-Range Chicken Farms. *Heilongjiang Animal Science and Veterinary Medicine* (2)**,** 71-72.

Li, C., Tie, F., and Cai, R. (2018a). Serological Antibody Testing of toxoplasma gondii in Yaks in Haiyan County, Qinghai. *Chinese Journal of Veterinary Medicine* 54(5)**,** 52-53.

Li, F., Wang, S.P., Wang, C.J., He, S.C., Wu, X., and Liu, G.H. (2016a). Seroprevalence of Toxoplasma gondii in goats in Hunan province, China. *Parasite* 23**,** 44. doi: 10.1051/parasite/2016053.

Li, G., Zheng, W., Yang, J., Qi, T., He, Y., Chen, W., et al. (2021). Seroprevalence and Epidemiology of Toxoplasma gondii in Animals in the Qinghai-Tibetan Plateau Area, China. *Pathogens* 10(4). doi: 10.3390/pathogens10040432.

Li, J. (2021). Investigation of Toxoplasma gondii Infection in Cattle. *China Animal Health* 23(8)**,** 37,47. doi: 10.3969/j.issn.1008-4754.2021.08.025.

Li, J., Zheng, B., Ren, H., Wang, S., Yao, Z., and Wand, D. (2018b). Investigation of Toxoplasma gondii Infection and Analysis of Risk Factors in Cattle in the Xinxiang Region of Henan. *Journal of Medical Pest Control* 34(10)**,** 922-924. doi: 10.7629/yxdwfz201810002.

Li, K., Gao, J., Shahzad, M., Han, Z., Nabi, F., Liu, M., et al. (2014). Seroprevalence of Toxoplasma gondii infection in yaks (Bos grunniens) on the Qinghai-Tibetan Plateau of China. *Vet Parasitol* 205(1-2)**,** 354-356. doi: 10.1016/j.vetpar.2014.07.014.

Li, M.H., Yang, B.T., Yin, Z.W., Wang, W., Zhao, Q., and Jiang, J. (2020). A Seroepidemiological Survey of Toxoplasma gondii and Chlamydia Infection in Chickens, Ducks, and Geese in Jilin Province, Northeastern China. *Vector Borne Zoonotic Dis* 20(11)**,** 825-830. doi: 10.1089/vbz.2020.2614.

Li, Q. (2022). Serological Detection of Toxoplasma gondii Antibodies in Large-Scale Dairy Cattle Farms. *Sichuan Animal & Veterinary Science* 49(8)**,** 21-23,26.

Li, Q., Qin, S.Y., Li, S., Peng, P., Zhao, Q., Jia, H.L., et al. (2019). First Report on Toxoplasma gondii Seroprevalence in Free-Ranging Pigs in Northeastern China. *Acta Parasitol* 64(2)**,** 295-299. doi: 10.2478/s11686-019-00045-9.

Li, W., Hou, X., Wang, Z., Han, M., Huang, Z., Pu, X., et al. (2018c). Epidemiological Survey of Toxoplasmosis in Large-Scale Sheep Herds in the Reclamation Area of Altay, Xinjiang. *China Animal Health Inspection* 35(3).

Li, X., Li, G., Jian, Y., Pei, Q., Li, P., and Ma, L. (2016b). Investigation and Research on Toxoplasmosis at Sanjiaocheng Sheep Breeding Farm in Qinghai Province. *Chinese Qinghai Journal of Animal and Veterinary Sciences* 46(3)**,** 14-15. doi: 10.3969/j.issn.1003-7950.2016.03.007.

Li, X., Xue, H., Wang, G., and Jin, L. (2017). Epidemiological Investigation of Toxoplasmosis in Hudong Sheep Farm, Qinghai Province. *Chinese Qinghai Journal of Animal and Veterinary Sciences* 47(1)**,** 45,44.

Li, Y. (2011). Serological Survey of Toxoplasmosis in Taosait Sheep and Their Offspring in Minhe County, Qinghai Province. *Anim Husb Feed Sci* (11)**,** 108-108. doi: 10.3969/j.issn.1672-5190.2011.11.059.

Li, Y., Li, Z., Kang, M., Li, Y., and Liu, L. (2010a). Serological Survey of Toxoplasma gondii in Tibetan Sheep in Huzhu County, Qinghai Province. *Progress in Veterinary Medicine* 31(7)**,** 119-121. doi: 10.3969/j.issn.1007-5038.2010.07.030.

Li, Y., Li, Z., Quan, H., Li, Y., and Hou, K. (2010b). Serological Survey of Toxoplasmosis in Yak in Huzhu County, Qinghai Province. *China Animal Health Inspection* 27(4)**,** 52-53. doi: 10.3969/j.issn.1005-944X.2010.04.026.

Li, Y.N., Nie, X., Peng, Q.Y., Mu, X.Q., Zhang, M., Tian, M.Y., et al. (2015). Seroprevalence and genotype of Toxoplasma gondii in pigs, dogs and cats from Guizhou province, Southwest China. *Parasit Vectors* 8**,** 214. doi: 10.1186/s13071-015-0809-2.

Liao, G., Liu, C., Xiao, J., Chen, M., Li, Y., and Chen, M. (2016). Serological Survey of Toxoplasmosis in Pigs in the Yongzhou Region of Hunan. *Chinese Journal of Animal Husbandry & Veterinary Medicine* (7)**,** 22-23. doi: 10.3969/j.Issn.1671-6027.2016.07.012.

Liao, Y. (2020). Serological investigation and analysis of swine toxoplasmosis in Miluo, Hunan Province from 2017 to 2019. *Swine Production* (6)**,** 105-106. doi: 10.3969/j.issn.1002-1957.2020.06.033.

Liu, F., Wang, D., Yang, S.C., Zhu, J.H., Li, J.M., Shi, K., et al. (2019a). Prevalence and Risk Factors of Brucellosis, Toxoplasmosis, and Neosporosis Among Yanbian Yellow Cattle in Jilin Province, China. *Vector Borne Zoonotic Dis* 19(3)**,** 217-221. doi: 10.1089/vbz.2018.2288.

Liu, H. (2014). Serological Survey of Toxoplasmosis in Pigs, Cattle, and Sheep in Wuping County. *Fujian Journal of Animal Husbandry & Veterinary Medicine* (2)**,** 16-17,18. doi: 10.3969/j.issn.1003-4331.2014.02.007.

Liu, J., Jiang, J., Li, X., Si, H., and Gu, Q. (2010a). Serological Investigation and Analysis of Toxoplasma gondii in Slaughter-Ready Pigs in Xinxiang City. *Mod Agric Sci Technol* (18)**,** 294,296. doi: 10.3969/j.issn.1007-5739.2010.18.182.

Liu, K. (2013). Serological Survey and Analysis of Toxoplasmosis in Pigs in Xinyang Region. *JOURNAL OF HENAN AGRICULTURAL SCIENCES* 42(5)**,** 166-168. doi: 10.3969/j.issn.1004-3268.2013.05.039.

Liu, L., Tian, Y., Zhang, J., Zhao, X., and J., L. (2015a). Serological Survey and Research on Toxoplasmosis in Pigs in Dazhou City. *China Animal Health* (9)**,** 72-74. doi: 10.3969/j.issn.1008-4754.2015.09.037.

Liu, L., Wang, G., Jiang, X., Miao, S., Wang, P., and Lu, G. (2019b). Monitoring and Analysis of Toxoplasma gondii Antibodies in Sheep in Selected Regions of Xinjiang. *Chinese Journal of Veterinary Medicine* 55(4)**,** 58-59.

Liu, L., Wang, P., Wang, J., Sha, Y., Jin, Y., and Lu, G. (2016). Monitoring and Analysis of Toxoplasma gondii Antibodies in Large-Scale Pig Farms in Certain Regions of Xinjiang. *Progress in Veterinary Medicine* 37(10)**,** 130-132. doi: 10.3969/j.issn.1007-5038.2016.10.029.

Liu, Q., Cai, J., Zhao, Q., Shang, L., Ma, R., Wang, X., et al. (2011). Seroprevalence of Toxoplasma gondii infection in yaks (Bos grunniens) in northwestern China. *Trop Anim Health Prod* 43(4)**,** 741-743. doi: 10.1007/s11250-010-9711-2.

Liu, Q., Ma, R., Zhao, Q., Shang, L., Cai, J., Wang, X., et al. (2010b). Seroprevalence of Toxoplasma gondii infection in Tibetan sheep in northwestern China. *J Parasitol* 96(6)**,** 1222-1223. doi: 10.1645/ge-2601.1.

Liu, S., and Zhu, J. (2017). Epidemiological Investigation of Toxoplasma gondii in Egg-Laying Chickens in the Dashiqiao Region of Liaoning Province. *Mod J Anim Husb Vet Med* (8)**,** 40-42.

Liu, X., Fan, L., Tan, Q., Chen, X., Li, H., Zhao, X., et al. (2022a). Prevalence of Toxoplasma gondii in pigs determined by ELISA based on recombinant SAG1 in Shandong province, China. *Comp Immunol Microbiol Infect Dis* 83**,** 101781. doi: 10.1016/j.cimid.2022.101781.

Liu, X., Kan, S., Li, R., Zhu, Z., Liang, X., and Lin, Q. (2015b). Serological Survey of Toxoplasma gondii in Dairy Goats at a Sheep Farm in Guanzhong Region. *Acta Agriculturae Boreali-occidentalis Sinica* 24(2)**,** 16-19. doi: 10.7606/j.issn.1004-1389.2015.02.004.

Liu, X., Liu, C., Liu, Y., Jin, H., Zhao, Y., Chen, J., et al. (2012). Seroprevalence of Toxoplasma gondii infection in slaughtered pigs and cattle in Liaoning Province, northeastern China. *J Parasitol* 98(2)**,** 440-441. doi: 10.1645/ge-2989.1.

Liu, X.C., He, Y., Han, D.G., Zhang, Z.C., Li, K., Wang, S., et al. (2017). Detection of Toxoplasma gondii in chicken and soil of chicken farms in Nanjing region, China. *Infect Dis Poverty* 6(1)**,** 62. doi: 10.1186/s40249-017-0277-3.

Liu, Y.M., Wang, L., Wang, H.Y., Li, C.H., Jiang, Y.H., and Sun, W.W. (2021). First detection of anti-Toxoplasma gondii antibodies in domestic goat's serum and milk during lactation in China. *Microb Pathog* 161(Pt B)**,** 105268. doi: 10.1016/j.micpath.2021.105268.

Liu, Y.M., Zhang, Y.Y., Wang, L., Wang, H.Y., Li, C.H., Jiang, Y.H., et al. (2022b). Toxoplasma gondii Antibodies in Raw Milk and Sera of Cows in China. *Pathogens* 11(10). doi: 10.3390/pathogens11101079.

Liu, Z.K., Li, J.Y., and Pan, H. (2015c). Seroprevalence and risk factors of Toxoplasma gondii and Neospora caninum infections in small ruminants in China. *Prev Vet Med* 118(4)**,** 488-492. doi: 10.1016/j.prevetmed.2014.12.017.

Lu, Y., Wang, G., Cai, Q., Ye, C., Niu, X., and Ma, L. (2012). Serological Survey of Toxoplasmosis in Yak at the Datong Cattle Breeding Farm in Qinghai Province. *China Animal Health Inspection* 29(3)**,** 40-41. doi: 10.3969/j.issn.1005-944X.2012.03.022.

Luo, H., Li, K., Zhang, H., Gan, P., Shahzad, M., Wu, X., et al. (2017). Seroprevalence of Toxoplasma gondii infection in zoo and domestic animals in Jiangxi Province, China. *Parasite* 24**,** 7. doi: 10.1051/parasite/2017007.

Luo, H.Q., Li, K., Zhang, H., Wu, B., Wang, J., Shahzad, M., et al. (2016). Seroepidemiology of Toxoplasma gondii and Neospora caninum infections in goats in Hubei province, China. *Trop Biomed* 33(2)**,** 285-289.

Lv, J., Gao, L., Zhang, D., and Pu, Z. (2014). Serological Testing for Toxoplasmosis in Semi-Wild Yak in Tianjun County. *Chinese Qinghai Journal of Animal and Veterinary Sciences* 44(3)**,** 22.

Lv, Q.Y., Quan, M.X., Tang, H.L., Wu, X.T., Liu, G.H., Li, F., et al. (2021). Seroprevalence, Risk Factors, and Genotypes of Toxoplasma gondii in Free-Range Chickens Intended for Human Consumption in China. *Foodborne Pathog Dis* 18(4)**,** 253-259. doi: 10.1089/fpd.2020.2844.

M, Q., L, D., J, Y., H, W., J, L., W, Z., et al. (2018). Serological Survey of Toxoplasma gondii in Sows in Large-Scale Pig Farms. *Heilongjiang Animal Science and Veterinary Medicine* (6)**,** 93-95. doi: 10.13881/j.cnki.hljxmsy.2017.07.0154.

M, Y., J, W., Q, T., S, Q., G, L., X, Z., et al. (2015). Serological Epidemiological Investigation and Risk Factor Analysis of Toxoplasma gondii in Yaks in Maqu County, Gansu Province. *Animal Husbandry & Veterinary Medicine* 47(10)**,** 105-108.

Ma, L., Li, S., Zhang, Y., and Wen, Z. (2021). Seroprevalence of Toxoplasma gondii and Neospora caninum in dairy cows in Hebei province, China. *Anim Biotechnol* 32(4)**,** 451-453. doi: 10.1080/10495398.2020.1714636.

Ma, L., Wang, Z.D., Li, J.P., Wei, F., and Liu, Q. (2015). Seroprevalence of Toxoplasma gondii infection in freerange chickens in Jilin Province, northeastern China. *Trop Biomed* 32(4)**,** 693-698.

Ma, S. (2020). Serological Survey and Analysis of Toxoplasmosis in Goats in Gaochun District. *Shanghai Journal of Animal Husbandry & Veterinary Medicine* (4)**,** 43,50.

Mao, K., Lin, Z., Lin, X., Lin, Z., Chen, Y., and Wu, D. (2013). Serological Survey of Chlamydiosis, Toxoplasmosis, and Brucellosis in Goats in Fuqing. *Fujian Journal of Animal Husbandry & Veterinary Medicine* (5)**,** 21-22. doi: 10.3969/j.issn.1003-4331.2013.05.008.

Meng, R. (2018). Serological Survey of Toxoplasmosis in Dairy Cattle in Xinchai County, Henan Province. *China Dairy* (6)**,** 46-47.

Ou, Y., Xu, C., Xue, T., Yang, Y., Lv, R., Song, H., et al. (2014). Serological Survey of Toxoplasmosis in Goats in Yuxi City. *Yunnan Journal of Animal Science and Veterinary Medicine* (3)**,** 12-13.

Pan, Y. (2019). Serological Survey and Analysis of Toxoplasmosis in Selected Pig Farms in Luohe City. *Rural Technology* (2)**,** 104-105. doi: 10.3969/j.issn.1674-7909.2019.02.054.

Qin, S.Y., Zhou, D.H., Cong, W., Zhang, X.X., Lou, Z.L., Yin, M.Y., et al. (2015). Seroprevalence, risk factors and genetic characterization of Toxoplasma gondii in free-range white yaks (Bos grunniens) in China. *Vet Parasitol* 211(3-4)**,** 300-302. doi: 10.1016/j.vetpar.2015.05.015.

Qiu, J.H., Wang, C.R., Zhang, X., Sheng, Z.H., Chang, Q.C., Zhao, Q., et al. (2012). Seroprevalence of Toxoplasma gondii in beef cattle and dairy cattle in northeast China. *Foodborne Pathog Dis* 9(7)**,** 579-582. doi: 10.1089/fpd.2011.1104.

Ren, Q., Zhang, X., and Li, W. (2011). Serological Survey of Toxoplasmosis in Some Townships of Tianshui County. *Chinese Qinghai Journal of Animal and Veterinary Sciences* 41(4)**,** 32-32. doi: 10.3969/j.issn.1003-7950.2011.04.023.

Ren, X. (2012). Serological Survey of Toxoplasmosis in Yaks in Hualong County, Qinghai Province, China. *Shandong Journal of Animal Science and Veterinary Medicine* 33(07)**,** 68-69.

Ren, X. (2022). Investigation of Subclinical Toxoplasma gondii Infection in Pigs in Coastal Areas of Shandong Province. *Shandong Journal of Animal Science and Veterinary Medicine* 43(7)**,** 16-17,20. doi: 10.3969/j.issn.1007-1733.2022.07.006.

Shao, J. (2017). Serological Survey and Analysis of Toxoplasmosis in Some Pig Farms in Xiamen City. *Fujian Journal of Animal Husbandry & Veterinary Medicine* 39(3)**,** 12-13. doi: 10.3969/j.issn.1003-4331.2017.03.005.

Su, R., Jiang, N., Lu, Y., Jian, F., Wang, H., Zhang, G., et al. (2020). Low prevalence of viable Toxoplasma gondii in swine from slaughter houses in the central of China. *Parasitol Int* 76**,** 102090. doi: 10.1016/j.parint.2020.102090.

Sun, C., Xia, B., Zhang, L., and Li, H. (2018). Serological Epidemiological Survey of Porcine Toxoplasma gondii in Border Areas of Yunnan Province. *Heilongjiang Animal Science and Veterinary Medicine* (8)**,** 100-101. doi: 10.13881/j.cnki.hljxmsy.2017.12.0278.

Sun, H. (2016). Serological Survey of Porcine Toxoplasmosis in Xinyang City, Henan Province. *Progress in Veterinary Medicine* 37(9)**,** 123-124,125. doi: 10.3969/j.issn.1007-5038.2016.09.028.

Sun, H., Zhang, Y., Zhang, L., Zhao, S., and Ma, C. (2016). Serological Survey and Analysis of Toxoplasmosis in Chickens in the Xinyang Region. *China Poultry Science* (6)**,** 48-49. doi: 10.3969/j.issn.1673-1085.2016.06.018.

Sun, L.X., Liang, Q.L., Nie, L.B., Hu, X.H., Li, Z., Yang, J.F., et al. (2020). Serological evidence of Toxoplasma gondii and Neospora caninum infection in black-boned sheep and goats in southwest China. *Parasitol Int* 75**,** 102041. doi: 10.1016/j.parint.2019.102041.

Sun, T., Rahman, S.U., Cai, J., Zeng, J., Mi, R., Zhang, Y., et al. (2021). Seroprevalence and associated risk factors of Toxoplasma gondii infection in yaks (Bos grunniens) on the Qinghai-Tibetan Plateau of China. *Parasite* 28**,** 43. doi: 10.1051/parasite/2021043.

Sun, W.W., Meng, Q.F., Cong, W., Shan, X.F., Wang, C.F., and Qian, A.D. (2015). Herd-level prevalence and associated risk factors for Toxoplasma gondii, Neospora caninum, Chlamydia abortus and bovine viral diarrhoea virus in commercial dairy and beef cattle in eastern, northern and northeastern China. *Parasitol Res* 114(11)**,** 4211-4218. doi: 10.1007/s00436-015-4655-0.

Tan, Q.D., Yang, X.Y., Yin, M.Y., Hu, L.Y., Qin, S.Y., Wang, J.L., et al. (2015). Seroprevalence and correlates of Toxoplasma gondii infection in dairy cattle in northwest China. *Acta Parasitol* 60(4)**,** 618-621. doi: 10.1515/ap-2015-0087.

Tang, W., Li, F., and Zhou, Y. (2022). Investigation of Toxoplasmosis Infection in Chickens in Selected Areas of Hunan Province. *Jiangxi Journal of Animal Husbandry & Veterinary Medicine* (6)**,** 26-27. doi: 10.3969/j.issn.1004-2342.2022.06.009.

Tang, W., Nie, F., Zhang, Y., Jia, Z., and Yin, F. (2020). Investigation of Toxoplasma gondii Infection in White Yaks in Tianzhu County. *China Herbivore Science* 40(3)**,** 91-92. doi: 10.3969/j.issn.2095-3887.2020.03.021.

Tang, X., Wang, C., Lin, Y., He, S., Wang, W., Lu, X., et al. (2018). Serological Survey of Toxoplasma gondii in Goats in Selected Areas of Hunan Province. *China Animal Health Inspection* 35(10)**,** 31-34. doi: 10.3969/j.issn.1005-944X.2018.10.009.

Tao, Q., Wang, Z., Feng, H., Fang, R., Nie, H., Hu, M., et al. (2011). Seroprevalence and risk factors for Toxoplasma gondii infection on pig farms in central China. *J Parasitol* 97(2)**,** 262-264. doi: 10.1645/ge-2646.1.

Tian, H., Wu, Z., Ren, Q., Yang, X., and Yang, Q. (2016). Serological Survey and Analysis of Brucellosis and Toxoplasmosis in White Goats in Yinjiang County. *Guizhou Journal of Animal Husbandry & Veterinary Medicine* 40(5)**,** 33-34. doi: 10.3969/j.issn.1007-1474.2016.05.010.

Tian, P., and Cui, P. (2010). Investigation of Avian Toxoplasma gondii Infection in Chickens in the Baxia Area of Zhangjiakou. *Anim Husb Feed Sci* 31(9)**,** 172-173. doi: 10.3969/j.issn.1672-5190.2010.09.094.

Wang, D., Liu, Y., Jiang, T., Zhang, G., Yuan, G., He, J., et al. (2016a). Seroprevalence and genotypes of Toxoplasma gondii isolated from pigs intended for human consumption in Liaoning province, northeastern China. *Parasit Vectors* 9**,** 248. doi: 10.1186/s13071-016-1525-2.

Wang, H., Fan, Z., Huang, J., Deng, G., Liu, D., Wang, C., et al. (2012a). Surveillance on toxoplasmosis of large-scale goat farm in Taojiang County in 2011. *Hunan Animal science and Veterinary Medicine* (6)**,** 26-28. doi: 10.3969/j.issn.1006-4907.2012.06.011.

Wang, J., Shi, D., Cheng, H., and Huo, J. (2013). Serological Survey of Toxoplasmosis in Dairy Cattle in Zhengzhou City. *Chinese Journal of Veterinary Medicine* 49(9)**,** 39-40. doi: 10.3969/j.issn.0529-6005.2013.09.014.

Wang, L., Zhao, G., Zhao, J., Gao, Y., Yan, S., Li, Y., et al. (2020). Detection of Trichinella and Toxoplasma gondii infection in Slaughtered Pigs in Selected Regions of China in 2019. *China Animal Health Inspection* 37(5)**,** 1-4,22. doi: 10.3969/j.issn.1005-944X.2020.05.001.

Wang, M. (2011). Serological Detection of Brucellosis, Chlamydia, and Toxoplasmosis in Cashmere Goats. *Journal of Domestic Animal Ecology* 32(6)**,** 80-81,123. doi: 10.3969/j.issn.1673-1182.2011.06.017.

Wang, M., Wang, Y.H., Ye, Q., Meng, P., Yin, H., and Zhang, D.L. (2012b). Serological survey of Toxoplasma gondii in Tibetan mastiffs (Canis lupus familiaris) and yaks (Bos grunniens) in Qinghai, China. *Parasit Vectors* 5**,** 35. doi: 10.1186/1756-3305-5-35.

Wang, M., Yin, H., Wang, S., Wang, S., Ma, Y., and Zhang, D. (2015). Epidemiological Investigation of Toxoplasmosis in Yaks in Tianzhu Region, Gansu. *Chin Dairy Cattl* (22)**,** 23-25. doi: 10.3969/j.issn.1004-4264.2015.22.008.

Wang, Q., Cheng, R., Zhao, Y., Liu, D., Zhou, X., Meng, K., et al. (2018a). The Prevalence and Analysis of Three Abortive Diseases in Cattle and Sheep in Haixi Prefecture. *Chin J Vet Drug* 52(7)**,** 1-7. doi: 10.11751/issn.1002-1280.2018.07.01.

Wang, Q., Huang, Y., Zhang, W., Sun, H., Chen, Y., Ku, E., et al. (2016b). Serological Survey of Toxoplasmosis in a Large-Scale Sheep Farm in Southern Xinjiang. *Anim Husb Feed Sci* 37(3)**,** 107-108,109. doi: 10.3969/j.issn.1672-5190.2016.03.040.

Wang, S., Li, X., and Ma, L. (2019a). The Survey of Bovine Toxoplasmosis in Jiaoding Town, Huzhu. *Chinese Qinghai Journal of Animal and Veterinary Sciences* 49(4)**,** 46-47. doi: 10.3969/j.issn.1003-7950.2019.04.009.

Wang, W. (2012). Serological Survey of Toxoplasmosis in Yaks in Huangyuan County, Qinghai Province. *Chinese Qinghai Journal of Animal and Veterinary Sciences* 42(3)**,** 26-26. doi: 10.3969/j.issn.1003-7950.2012.03.020.

Wang, Y., Chang, L., Zhang, L., Wang, F., Wang, P., and Wang, S. (2018b). Antibody Detection of Toxoplasma gondii Infection in Sows from Some Pig Farms in Xuzhou City, Jiangsu Province. *China Animal Health Inspection* 35(9)**,** 19-22. doi: 10.3969/j.issn.1005-944X.2018.09.006.

Wang, Y.G., Gui, B.Z., Li, R.C., Wang, G.P., Ge, M., and Liu, G.H. (2019b). Seroprevalence and Risk Factors of Toxoplasma gondii Infection in Growth Stages of Pigs in Hunan Province, Subtropical China. *Vector Borne Zoonotic Dis* 19(12)**,** 945-949. doi: 10.1089/vbz.2019.2464.

Wang, Z., Li, K., Sun, Y., Li, H., Yang, H., Dong, C., et al. (2018c). Seroepidemiological Survey and Risk Factor Analysis of Bovine Toxoplasmosis in Certain Areas of Chongqing. *Journal of Southwest University (Natural Science)* 40(5)**,** 8-12. doi: 10.13718/j.cnki.xdzk.2018.05.002.

Wen, Q., Guo, Y., Yang, J., Shen, H., and Du, Y. (2015). Serological Survey of Toxoplasma Gondii Infection in Pigs in Henan Province. *Chinese Journal of Veterinary Medicine* 51(4)**,** 44-45. doi: 10.3969/j.issn.0529-6005.2015.04.014.

Wu, D., Lv, R., Sun, X., Shu, F., Zhou, Z., Nie, K., et al. (2012a). Seroprevalence of Toxoplasma gondii antibodies from slaughter pigs in Chongqing, China. *Trop Anim Health Prod* 44(4)**,** 685-687. doi: 10.1007/s11250-011-9965-3.

Wu, F., Wang, Y.L., Yang, Z., Li, X.L., Li, Z.R., and Lin, Q. (2017). Seroprevalence and Risk Factors of Toxoplasma gondii in Slaughter Pigs in Shaanxi Province, Northwestern China. *Vector Borne Zoonotic Dis* 17(7)**,** 517-519. doi: 10.1089/vbz.2016.2103.

Wu, S., Zhao, D., Sun, F., Zhao, Y., Xue, S., and Xu, Y. (2018). Preliminary Serological Survey of Toxoplasma gondii in Chickens in the Yanbian Area. *Heilongjiang Animal Science and Veterinary Medicine* (11)**,** 103-104. doi: 10.13881/j.cnki.hljxmsy.2018.03.0242.

Wu, S.M., Ciren, D., Huang, S.Y., Xu, M.J., Ga, G., Yan, C., et al. (2012b). First report of Toxoplasma gondii prevalence in Tibetan pigs in Tibet, China. *Vector Borne Zoonotic Dis* 12(8)**,** 654-656. doi: 10.1089/vbz.2012.0968.

Wu, S.M., Danba, C., Huang, S.Y., Zhang, D.L., Chen, J., Gong, G., et al. (2011). Seroprevalence of Toxoplasma gondii infection in Tibetan sheep in Tibet, China. *J Parasitol* 97(6)**,** 1188-1189. doi: 10.1645/ge-2912.1.

Xiang, Z., Duan, Y., Yue, X., and Chen, J. (2011). Serological Survey and Study of Porcine Toxoplasmosis in Some Areas of Anhui. *Hunan Agricultural Sciences* (17)**,** 134-135,138. doi: 10.3969/j.issn.1006-060X.2011.17.040.

Xie, W., Qiao, S., Liang, Y., Hao, H., Deng, R., Zhang, L., et al. (2019). Sero-epidemiological investigation of Toxoplasma gondii in scaled pig farms of Henan province in 2017-2018. *Chinese Journal of Veterinary Science* 39(11)**,** 2179-2183,2189. doi: 10.16303/j.cnki.1005-4545.2019.11.15.

Xing, D., Zhang, X., Duo, H., and Guo, Z. (2022). Serological Survey of Toxoplasma gondii in Tibetan Sheep in Selected Regions of Qinghai Province. *Chinese Qinghai Journal of Animal and Veterinary Sciences* 52(6)**,** 42-43. doi: 10.3969/j.issn.1003-7950.2022.06.008.

Xu, B., Zhang, X., Dong, C., Feng, C., Su, B., He, Q., et al. (2013). Epidemiological Investigation and Research on Porcine Toxoplasmosis in the Chongqing Region. *Progress in Veterinary Medicine* 34(3)**,** 37-40. doi: 10.3969/j.issn.1007-5038.2013.03.009.

Xu, M.J., Liu, Q.Y., Fu, J.H., Nisbet, A.J., Shi, D.S., He, X.H., et al. (2012a). Seroprevalence of Toxoplasma gondii and Neospora caninum infection in dairy cows in subtropical southern China. *Parasitology* 139(11)**,** 1425-1428. doi: 10.1017/s0031182012000728.

Xu, P., Cai, Y.N., Leng, X., Wang, J., Ma, W., Mu, G.D., et al. (2015a). Seroprevalence of Toxoplasma gondii infection in pigs in Jilin Province, Northeastern China. *Trop Biomed* 32(1)**,** 116-120.

Xu, P., Li, X., Guo, L., Li, B., Wang, J., Yu, D., et al. (2014a). Seroprevalence of Toxoplasma gondii infection in Liaoning cashmere goat from northeastern China. *Parasite* 21**,** 22. doi: 10.1051/parasite/2014023.

Xu, P., Li, X., Tang, F., Liu, Y.H., Kou, X., Zhao, M.L., et al. (2015b). Seroprevalence and risk factors for Toxoplasma gondii in sheep and goats in Jinzhou, Northeastern China. *Trop Biomed* 32(3)**,** 563-567.

Xu, P., Song, X., Wang, W., Wang, F., Cao, L., and Liu, Q. (2012b). Seroprevalence of Toxoplasma gondii infection in chickens in Jinzhou, northeastern China. *J Parasitol* 98(6)**,** 1300-1301. doi: 10.1645/ge-3164.1.

Xu, Y., Li, R.C., Liu, G.H., Cong, W., Zhang, X.X., Yu, X.L., et al. (2014b). Seroprevalence of Toxoplasma gondii infection in sows in Hunan province, China. *ScientificWorldJournal* 2014**,** 347908. doi: 10.1155/2014/347908.

Xu, Z., Ming, W., Liu, M., Chen, Q., and Ba, Y. (2016). Serological Testing for Swine Toxoplasmosis in Bazhou Area, Xinjiang. *Animal Husbandry & Veterinary Medicine* 48(9)**,** 114-115.

Yan, P., Shang, X., Tian, Z., and Hua, L. (2020a). Serological Survey of Toxoplasma gondii in Large-Scale Pig Farms in Zhejiang and Hebei Provinces. *Mod J Anim Husb Vet Med* (1)**,** 50-53.

Yan, X., Han, W., Han, X., Luo, S., and Wang, Y. (2021). Serological Survey of Ovine Toxoplasmosis in Central and Western Inner Mongolia. *Chinese Journal of Veterinary Medicine* 57(1)**,** 44-46.

Yan, X., Han, W., Wang, Y., Zhang, H., and Gao, Z. (2020b). Seroprevalence of Toxoplasma gondii infection in sheep in Inner Mongolia Province, China. *Parasite* 27**,** 11. doi: 10.1051/parasite/2020008.

Yang, M., Yang, P., Xiao, J., Wen, Z., and Hu, Z. (2011). Serological Survey of Swine Toxoplasmosis in Wuding County. *Technical Advisor for Animal Husbandry* (10)**,** 87-88. doi: 10.3969/j.issn.1673-1921.2011.10.081.

Yang, N., Li, H., He, J., Mu, M., and Yang, S. (2013). Seroprevalence of Toxoplasma gondii infection in domestic sheep in Liaoning Province, northeastern China. *J Parasitol* 99(1)**,** 174-175. doi: 10.1645/ge-3201.1.

Yang, N., Mu, M.Y., Li, H.K., Long, M., and He, J.B. (2012). Seroprevalence of Toxoplasma gondii infection in slaughtered chickens, ducks, and geese in Shenyang, northeastern China. *Parasit Vectors* 5**,** 237. doi: 10.1186/1756-3305-5-237.

Yang, N., Xing, M., Wang, D., and Wu, Y. (2017a). Isolation and Identification of Toxoplasma gondii Strains from Swine Sources in Liaoning Province. *J Huazhong Agric Univ* 36(2)**,** 84-88.

Yang, Q., and Shi, X. (2023). Serological Survey of Toxoplasmosis in Pig Farms in Bijiang District, Tongren City, from 2021 to 2022. *Yunnan Journal of Animal Science and Veterinary Medicine* (2)**,** 12-14. doi: 10.3969/j.issn.1005-1341.2023.02.005.

Yang, X., and Zhao, G. (2015). Investigation and Analysis of Toxoplasma gondii Infection in Pigs at Different Rearing Stages in the Chongqing Region. *Chinese Journal of Veterinary Medicine* 51(2)**,** 44-45. doi: 10.3969/j.issn.0529-6005.2015.02.015.

Yang, Z., Mo, Z., and Huang, H. (2017b). Epidemiological Investigation and Analysis of Swine Toxoplasma gondii in Dongguan from 2010 to 2016. *Rural Economy and Science-Technology* 28(14)**,** 44-45. doi: 10.3969/j.issn.1007-7103.2017.14.035.

Yin, M.Y., Wang, J.L., Huang, S.Y., Qin, S.Y., Zhou, D.H., Liu, G.X., et al. (2015a). Seroprevalence and risk factors of Toxoplasma gondii in Tibetan Sheep in Gansu province, Northwestern China. *BMC Vet Res* 11**,** 41. doi: 10.1186/s12917-015-0358-0.

Yin, Y., Li, G., Wu, Y., Dong, B., Wang, J., and Meng, B. (2015b). Serological Survey of Porcine Toxoplasmosis in Qiannan Prefecture. *Contemporary Animal Husbandry* (11)**,** 81-82.

You, J., Zhang, R., Qiu, J., Xie, M., and Hu, L.Y. (2015). Epidemiological Survey Report on Swine Toxoplasmosis in Fujian Province. *Contemporary Animal Husbandry* (2)**,** 22-23.

Yu, B., Yin, C., Yu, X., Ai, M., Li, X., Xue, S., et al. (2018). Preliminary Investigation of Bovine Toxoplasma Infection in Yanbian Area. *Agriculture of Jilin* (1)**,** 70-71. doi: 10.14025/j.cnki.jlny.2018.01.033.

Yu, H.J., Zhang, Z., Liu, Z., Qu, D.F., Zhang, D.F., Zhang, H.L., et al. (2011). Seroprevalence of Toxoplasma gondii infection in pigs, in Zhejiang Province, China. *J Parasitol* 97(4)**,** 748-749. doi: 10.1645/ge-2713.1.

Zhang, B., Guan, Y., Li, Y., Liu, P., and Tao, J. (2018). Serological Epidemiological Survey of Toxoplasma gondii in Swine in Taizhou Region. *Chinese Journal of Veterinary Medicine* 54(11)**,** 13-15.

Zhang, H., A, S., Cang, N., Hou, H., Zhou, M., Ka, D., et al. (2014). Serological Survey of Toxoplasmosis in Cattle and Sheep in Zeku County, Qinghai Province. *Animal Husbandry & Veterinary Medicine* 46(8)**,** 125-126.

Zhang, N., Wang, S., Wang, D., Li, C., Zhang, Z., Yao, Z., et al. (2016). Seroprevalence of Toxoplasma gondii infection and risk factors in domestic sheep in Henan province, central China. *Parasite* 23**,** 53. doi: 10.1051/parasite/2016064.

Zhang, X., Li, W., and Lu, Y. (2010). Serological Survey and Analysis of Toxoplasmosis in Yaks in Tianjun County, Qinghai Province. *Animal Husbandry & Veterinary Medicine* 42(1)**,** 107-108.

Zhang, Y., Gong, H., Mi, R., Huang, Y., Han, X., Xia, L., et al. (2020). Seroprevalence of Toxoplasma gondii infection in slaughter pigs in Shanghai, China. *Parasitol Int* 76**,** 102094. doi: 10.1016/j.parint.2020.102094.

Zhang, Y., Mi, R., Xie, J., Jia, H., Ling, H., Zhang, X., et al. (2022). Seroprevalence and the Risk Factor of Toxoplasma gondii Infection to Slaughter Pigs in Chongqing, China. *Vector Borne Zoonotic Dis* 22(4)**,** 238-243. doi: 10.1089/vbz.2021.0101.

Zhao, G., Shen, B., Xie, Q., Xu, L.X., Yan, R.F., Song, X.K., et al. (2012). Detection of Toxoplasma gondii in free-range chickens in China based on circulating antigens and antibodies. *Vet Parasitol* 185(2-4)**,** 72-77. doi: 10.1016/j.vetpar.2011.10.031.

Zhao, G.H., Zhang, M.T., Lei, L.H., Shang, C.C., Cao, D.Y., Tian, T.T., et al. (2011a). Seroprevalence of Toxoplasma gondii infection in dairy goats in Shaanxi Province, Northwestern China. *Parasit Vectors* 4**,** 47. doi: 10.1186/1756-3305-4-47.

Zhao, L. (2015a). Serological Survey of Toxoplasmosis in Tibetan Sheep in Xinghai County. *Chinese abstracts of Animal Husbandry and Veterinary Medicine* (8)**,** 115-115.

Zhao, L. (2015b). Serological Survey of Toxoplasmosis in Yaks in High-Cold and High-Altitude Regions. *Veterinary Orientation* (14)**,** 56. doi: 10.3969/j.issn.1673-8586.2015.14.049.

Zhao, P., Zhang, S., Jia, L., Yu, L., Li, N., and Li, J. (2016). Serological Survey of Bovine Toxoplasmosis in Changchun Area, Jilin Province. *Animal Husbandry & Veterinary Medicine* 48(5)**,** 146-147.

Zhao, Q., Hu, G., Li, J., Li, L., Ma, Z., Yuan, Y., et al. (2011b). Serological Survey of Ovine Toxoplasmosis in Delingha Area, Qinghai Province. *Shanghai Journal of Animal Husbandry & Veterinary Medicine* (2)**,** 51. doi: 10.3969/j.issn.1000-7725.2011.02.024.

Zhao, Z., He, Q., Yang, J., Li, G., Gu, D., Zhang, C., et al. (2018). Serological Survey of Toxoplasmosis in Cattle and Sheep in Selected Townships of Datong County. *Chinese Qinghai Journal of Animal and Veterinary Sciences* 48(6)**,** 46,42. doi: 10.3969/j.issn.1003-7950.2018.06.011.

Zheng, B., Li, J., Ren, H., Wang, S., Zhang, H., and Yao, Z. (2017a). Serological Investigation of Toxoplasma Infection in Chickens in the Xinxiang Area, Henan Province, from 2011 to 2013. *Journal of Medical Pest Control* 33(5)**,** 476-478. doi: 10.7629/yxdwfz201705002.

Zheng, B., Yin, Z., Li, J., Yao, Z., Wang, S., Ren, H., et al. (2017b). Analysis of Toxoplasma gondii Infection and Risk Factors in Swine in the Xinxiang Area, Henan Province. *Progress in Veterinary Medicine* 38(6)**,** 117-119.

Zheng, L., and Feng, G. (2019). Detection and Analysis of Infectious Antibodies for Toxoplasmosis in Goats in Wulong. *Livestock and Poultry Industry* 30(6)**,** 11-12. doi: 10.19567/j.cnki.1008-0414.2019.06.005.

Zhou, D.H., Liang, R., Yin, C.C., Zhao, F.R., Yuan, Z.G., Lin, R.Q., et al. (2010). Seroprevalence of Toxoplasma gondii in pigs from southern China. *J Parasitol* 96(3)**,** 673-674. doi: 10.1645/ge-2416.1.

Zhou, D.H., Zhao, F.R., Lu, P., Xia, H.Y., Xu, M.J., Yuan, L.G., et al. (2012). Seroprevalence of Toxoplasma gondii infection in dairy cattle in southern China. *Parasit Vectors* 5**,** 48. doi: 10.1186/1756-3305-5-48.

Zhou, X. (2018). Serological Survey and Analysis of Toxoplasma gondii in Sheep in Baofeng County. *Henan Journal of Animal Husbandry and Veterinary Medicine* 39(11)**,** 32-33.

Zhou, X., Zhou, H., Ning, X., Li, J., Jian, F., Zhang, L., et al. (2014). Serological Epidemiological Survey of Toxoplasma gondii in Cattle and Sheep in Selected Regions of China. *China Herbivore Science* (5)**,** 43-45,46. doi: 10.3969/j.issn.2095-3887.2014.05.013.

Zhou, Y., Hao, F., Yu, J., Sun, M., Zhang, T., Su, M., et al. (2022). Investigation of Porcine Toxoplasma gondii Infection in Pig Farms Surrounding Jilin City and Preliminary Genotype Identification of Isolates. *Heilongjiang Animal Science and Veterinary Medicine* (3)**,** 72-76,136. doi: 10.13881/j.cnki.hljxmsy.2021.01.0222.

Zhou, Z., Wu, Y., Chen, Y., Wang, Z., Hu, S., Zhou, R., et al. (2018). Molecular and serological prevalence of Toxoplasma gondii and Anaplasma spp. infection in goats from Chongqing Municipality, China. *Parasite* 25**,** 20. doi: 10.1051/parasite/2018024.

Zou, F., Yu, X., Yang, Y., Hu, S., Chang, H., Yang, J., et al. (2015). Seroprevalence and Risk Factors of Toxoplasma gondii Infection in Buffaloes, Sheep and Goats in Yunnan Province, Southwestern China. *Iran J Parasitol* 10(4)**,** 648-651.
